# Targeted therapies for myocardial infarction based on COPD-related extracellular vesicles

**DOI:** 10.1038/s41598-026-50402-8

**Published:** 2026-05-11

**Authors:** Zhao Gao, Haiyan Wang

**Affiliations:** Department of Cardiology, The First Affiliated Hospital of Xi’an Jiaotong University, Xi’an, China

**Keywords:** Chronic obstructive pulmonary disease, Extracellular vesicles, Myocardial infarction, PI3K/Akt signaling pathway, Mitochondrial dysfunction, Cardiology, Cell biology, Diseases, Medical research

## Abstract

Chronic obstructive pulmonary disease (COPD) is a significant independent risk factor for myocardial infarction (MI), leading to impaired cardiac repair and poor prognosis. Small extracellular vesicles (sEVs), a key subtype of extracellular vesicles, are crucial mediators of inter-organ communication and may participate in harmful cardiopulmonary crosstalk. However, the net impact of COPD-context sEVs on post-MI injury and repair remains unclear. Here, we generated COPD-mimetic extracellular vesicles from cigarette smoke extract (CSE)-stimulated bronchial epithelial cells and investigated their effects and mechanisms in ischemic myocardial injury. COPD-mimetic extracellular vesicles (CSE-sEVs) were isolated from conditioned medium of CSE-stimulated BEAS-2B bronchial epithelial cells and characterized by transmission electron microscopy, nanoparticle tracking analysis, zeta potential measurement, and sEVs marker immunoblotting (CD63, CD81, TSG101). Cardiomyocyte injury models were treated with different graded sEVs doses in vitro, followed by assessment of cell viability, oxidative stress (SOD/MDA, ROS), inflammatory cytokines (IL-6, TNF-α, IL-1β), migration/invasion, mitochondrial membrane potential (JC-1), and apoptosis. Furthermore, MI mice received sEVs in vivo, and cardiac function and remodeling were evaluated by echocardiography and histology. Mechanistic dependency on PI3K/Akt signaling was tested using the selective inhibitor LY294002. COPD-related extracellular vesicles displayed canonical morphology, size distribution, and marker enrichment, with high colloidal stability. Extracellular vesicles treatment dose-dependently improved cardiomyocyte viability, suppressed ROS and pro-inflammatory cytokine release, restored mitochondrial membrane potential, and reduced apoptosis accompanied by downregulation of Bax/NF-κB/COX-2 and upregulation of Bcl-2. In MI mice, sEVs administration improved systolic function, attenuated ventricular dilation, mitigated myocardial injury, and reduced fibrotic remodeling. Pharmacologic blockade of PI3K/Akt with LY294002 substantially abrogated extracellular vesicles-mediated pro-survival, mitochondrial, anti-apoptotic, and antioxidant effects, supporting a PI3K/Akt-centered mechanism. COPD-mimetic sEVs confer robust cardioprotection after ischemic injury, in part by activating PI3K/Akt signaling to improve mitochondrial integrity while restraining NF-κB-linked inflammation, oxidative stress, and apoptosis. PI3K/Akt signaling contributes to, but may not fully account for, the observed effects.

**Supplementary Information:**

The online version contains supplementary material available at 10.1038/s41598-026-50402-8.

## Introduction

Chronic obstructive pulmonary disease (COPD) is a chronic inflammatory disorder characterized by persistent airflow limitation, and its development is strongly associated with long-term cigarette smoking, air pollution, and respiratory infections^1,2^. Growing evidence supports that small extracellular vesicles (sEVs) derived from different cellular sources exert heterogeneous biological effects in cardiovascular diseases^3–5^. Although COPD is characterized by chronic inflammation and many studies emphasize pro-inflammatory or tissue-damaging EV cargos, EV populations are heterogeneous and can also convey compensatory, tissue-protective signals in a context- and cell source–dependent manner. For example, cigarette smoke extract (CSE)-treated BEAS-2B bronchial epithelial cells release sEVs with reduced miR-21 that alleviate macrophage polarization and mitigate epithelial–mesenchymal transition, suggesting a compensatory epithelial EV program in COPD^6^. Moreover, airway basal stem cell–derived sEVs delivered to elastase-induced COPD mice reproduce the reparative effects of basal stem cells, promoting angiogenesis and epithelial repair; proteomic analyses implicate activation of the PI3K–Akt pathway^7,8^. In addition, endothelial progenitor cell -derived exosomes carrying miR-26a-5p suppress CSE-induced ferroptosis and EMT in bronchial epithelial cells and improve smoke-induced airway remodeling^9^. Smoke-exposed monocyte-derived exosomes have also been reported to transport protective messages that reduce cytotoxicity in recipient cells^10–12^. Collectively, these observations support the concept that COPD-context sEVs can adopt reparative phenotypes. However, whether and how such EV programs influence post-MI cardiac injury and remodeling remains insufficiently defined.

In this study, we used cigarette smoke extract (CSE)-stimulated bronchial epithelial cells to generate a COPD-mimetic sEVs preparation and investigated its impact on post-infarction cardiac repair. We hypothesized that COPD-associated stress can reshape sEVs cargo to engage pro-survival signaling in cardiomyocytes. We aimed to define how these extracellular vesicles modulate inflammation, apoptosis, and mitochondrial dysfunction following ischemic injury and to test the involvement of PI3K/Akt signaling using integrated in vitro and in vivo approaches, thereby providing a mechanistic basis for understanding cardiopulmonary crosstalk and informing mechanism-guided EV-based interventions for MI complicated by COPD.

## Materials and methods

### Cigarette smoke extract (CSE) preparation

CSE was prepared according to the method described previously^13^. Briefly, a non-filtered Furong cigarette (specifications: 14 mg carbon monoxide, 1.0 mg nicotine, and 13 mg tar per cigarette) was combusted, and the generated smoke was fully bubbled through 4 mL of phosphate-buffered saline (PBS) via a tubing system to obtain the stock CSE solution (defined as 100% concentration). Subsequently, the CSE solution was filtered through a 0.22 μm membrane filter and immediately used within 30 min of preparation. BEAS-2B (Newgainbio, Wuxi, China) were cultured in high-glucose-DMEM (Thermo Fisher Scientific, Waltham, MA, USA) containing 10% FBS (ScienCell, Carlsbad, CA, USA), 100 U/ml penicillin, and 100 µg/mL streptomycin under the condition of 37 °C and 5% CO_2_. CSE was added to the cell culture medium to a final concentration of 5%-10%, and the cells were treated under this condition for 24–48 h. All cells were authenticated and free of mycoplasma.

### Extraction and identification of extracellular vesicles

Extracellular vesicles were isolated from conditioned medium of CSE-stimulated BEAS-2B cells as previously described^14^. Briefly, conditioned medium was collected and cleared by sequential centrifugation at 4 °C (300 × g for 10–30 min, 2000 × g for 20–30 min, and 10,000 ×g for 30 min) to remove cells and debris. The supernatant was filtered through a 0.45 μm membrane and ultracentrifuged at 100,000 ×g for 70 min at 4 °C. The pellet was resuspended in pre-chilled PBS, filtered through a 0.22 μm membrane, and ultracentrifuged again at 100,000 ×g for 70 min. The final pellet was resuspended in 100–200 µL PBS and stored at − 80 °C until use. sEVs protein concentration was determined by a BCA assay (P0009, Beyotime), and doses for in vitro experiments were normalized to sEVs protein (10, 50, and 100 µg/mL). The protein dosage of 10 µg/mL used in our experiments corresponds to approximately 3 × 10^11^ particles/ml. Vesicle morphology was examined by transmission electron microscope (Hitachi H-7650, Japan). Particle size distribution and concentration were analyzed by NTA (ZetaView™ 120, Particle Metrix), and zeta potential was measured using a Zetasizer (Malvern). sEVs markers (CD63, CD81, and TSG101) were assessed by western blotting using anti-CD81 (ab79559, mouse monoclonal, 1:1,000, abcam), anti-CD63 (ab217345, rabbit monoclonal, 1:1,000, abcam), and anti-TSG101 (ab30871, rabbit monoclonal, 1:1,000, abcam) antibodies. Human Umbilical Vein Endothelial Cells (HUVECs) were used as a negative control for exosome marker validation. The cells were cultured in Endothelial Cell Medium (ECM) (ScienCell, Carlsbad, CA, USA) supplemented with 5% fetal bovine serum (FBS), 1% endothelial cell growth supplement (ECGS), and 1% penicillin/streptomycin solution. The cultures were maintained in a humidified incubator at 37 °C with 5% CO_2_. All cells were routinely tested and confirmed to be free of mycoplasma contamination.

### Cell culture

H9c2, rat cardiomyocytes were purchased from the Cell Bank of the Chinese Academy of Sciences (Shanghai, China). Cells were incubated under hypoxic conditions for 10 h followed by reoxygenation under normoxic conditions for 6 h. The cells were grouped into five groups: control group, model group, LD+sEVs group (Low Dose, 10 µg/mL), MD+sEVs group (Middle Dose, 50 µg/mL) and HD+sEVs group (High Dose, 100 µg/mL).

### Enzyme-linked immunosorbent assay (ELISA)

Cell culture supernatants were collected for subsequent cytokine analysis. ELISA kits were used to measure levels of TNF-α (SEKH-0047, Solarbio), IL-6 (SEKH-0013,Solarbio), IL-10 (BMS614-2, eBioscience), and IL-1β (SEKH-0002, Solarbio), following the manufacturer’s protocols.

### Cell counting kit-8 (CCK‐8) assay

Cell viability was assessed using the CCK-8 assay (C0037, Beyotime) following the manufacturer’s instructions. Briefly, cells were seeded in 96-well plates and treated with different conditions for 24 h. After incubation, 10 µL of CCK-8 solution was added to each well, followed by a 1.5-hour incubation. The absorbance was then measured at 450 nm using a microplate reader.

### Intracellular ROS detection

The fluorescent probe DCFH-DA (S1105S, Beyotime) was employed to track reactive oxygen species generation. The DCFH-DA probe was applied to treated cells for 20 min, followed by triple PBS washes. Intracellular ROS evaluation was performed by fluorescence microscopy following a 10-minute staining with Hoechst 33,342 (S1105S, Beyotime).

### Assessment of oxidative stress through SOD and MDA content analysis

Cells were harvested at different treatment groups and the levels of Malondialdehyde (MDA) and Superoxide dismutase (SOD) were measured using assay kits (BC0025, BC5165, Solarbio). The assays were performed according to the manufacturer’s instructions.

### JC-1-based evaluation of mitochondrial depolarization

Mitochondrial membrane potential assessment was performed using assay kits (C2006, Beyotime) by collecting cells in 24-well plates (4 × 10⁴ cells/well) at different treatment groups. Cells underwent PBS washing before incubation with 1 mL JC-1 solution (30 min). After removing excess dye through two washes, 1 mL serum-free medium was added. Finally, an inverted epifluorescence microscope was employed for dynamic observation of mitochondria in both J-monomers and J-aggregates.

### Migration assay

Following 12-hour serum starvation in DMEM, H9c2 cell migration was assessed using 8 μm pore Transwell inserts. Cells (2 × 10⁴/well) were suspended in serum-free medium in upper chambers, while complete medium filled lower chambers. After 24 h, fixed cells (4% PFA) were stained with 0.5% crystal violet (30 min), imaged by brightfield microscopy, and quantified using Image-J software.

### Invasion assay

Based on the migration assay, the Basement Membrane Matrix (C0383, Beyotime) was pre-coated in the upper chamber of the transwell, the rest of the operations were consistent with the migration assay.

### Cell apoptosis

Apoptosis was assessed using flow cytometry with annexin V-fluorescein isothiocyanate (FITC) and propidium iodide (PI) staining, following the manufacturer’s instructions (C1062S, Beyotime). Briefly, at the designated time points, cells were harvested, washed twice with cold PBS, and centrifuged at 1000 rpm for 5 min. The cells were then resuspended in 1× Annexin V Binding Buffer and incubated for 15 min. Subsequently, 5 µL of annexin V-FITC was added to 100 µL of cell suspension, followed by 5 µL of PI after 15 min of incubation. The cell apoptosis was analyzed using a FACS Calibur flow cytometer (BD).

### Animal

Male C57BL/6J mice aged 8–10 weeks (weighing 22–25 g) from Qinglong Mountain Animal Experimental Center were selected and housed under specific pathogen-free (SPF) conditions. The environmental temperature was maintained at 22 ± 2 °C, with a relative humidity of 50%–60% and a 12-hour light/dark alternating cycle. Mice had free access to food and water. All animal experimental protocols were approved by the Experimental Animal Ethics Committee of The First Affiliated Hospital of Xi’an Jiaotong University (Department of Cardiology, Xi’an, China, SCXK 2020-0009), and strictly complied with the *Regulations on the Administration of Experimental Animals* and the National Institutes of Health (NIH) Guidelines for the Care and Use of Laboratory Animals.

### Induction of MI model and intramyocardial injection of extracellular vesicles

Mice were anesthetized with 1%–2% isoflurane via inhalation, then fixed in a supine position and received endotracheal intubation for assisted ventilation. A left thoracotomy was performed through the 4th intercostal space; after exposing the heart, the left anterior descending coronary artery (LAD) was ligated with 6–0 silk suture to induce myocardial infarction. The criteria for successful surgery were the obvious pallor in the ischemic area and elevation of the ST segment on the electrocardiogram^15^. For mice in the sham operation group, only thread passing (around the LAD) was performed without ligation, while all other procedures were the same as those in the myocardial infarction group. Postoperatively, appropriate analgesic treatment and heat preservation care were provided to all mice.

The mice were randomized into five groups: control group (sham-operated + PBS, *n* = 3), model group (MI + PBS, *n* = 3), LD+sEVs group (Low Dose, 10 µg/kg, *n* = 3), MD+sEVs group (Middle Dose, 50 µg/kg, *n* = 3) and HD+sEVs group (High Dose,100 µg/kg, *n* = 3). Immediately after LAD ligation, extracellular vesicles suspended in PBS were injected into myocardium at four sites around the infarct border zone^16^. Mice were euthanized at 3 and 7 days after MI for tissue harvesting. At the designated endpoints, mice were euthanized by an overdose of inhaled isoflurane until deep anesthesia and respiratory arrest, followed by cervical dislocation as a secondary method to ensure death. Death was confirmed by absence of respiration and heartbeat prior to tissue harvesting.

### Assessment of cardiac function

Cardiac function was evaluated following a previously established protocol^17^. In brief, mice were anesthetized using a mixture of 2% isoflurane and oxygen, then positioned in a supine posture. For each mouse, M-mode echocardiographic images and grayscale two-dimensional parasternal short-axis views at the mid-papillary level were acquired. Subsequent offline analyses were conducted by a single investigator who was blinded to the experimental groups to avoid bias. From the M-mode images, two key dimensional parameters were quantified: left ventricular end-systolic diameter (LVESD) and left ventricular end-diastolic diameter (LVEDD). Additionally, left ventricular end-systolic volume (LVESV) and left ventricular end-diastolic volume (LVEDV) were measured to compute two primary indices of cardiac systolic function—left ventricular ejection fraction (LVEF) and left ventricular fractional shortening (FS)—using the formulas below:

$$\begin{aligned} {\text{LVEF }} & = {\text{ }}[({\mathrm{LVEDV}} - {\mathrm{LVESV}})/{\mathrm{LVEDV}}]{\text{ }} \times {\text{ 1}}00\% \\ {\text{LVFS }} & = {\text{ }}\left[ {\left( {{\mathrm{LVEDD}}\, - \,{\mathrm{LVESD}}} \right)/{\mathrm{LVEDD}}} \right]{\text{ }} \times {\text{ 1}}00\% \\ \end{aligned}$$

To ensure the reliability of the data, all echocardiographic measurements were repeated three times, with the operator remaining blinded to group assignments throughout the entire process.

### RNA isolation and reverse transcription polymerase chain reaction (RT-PCR) analysis

The total RNAs were extracted with TRIzol reagent (Nanjing Novizan Biotechnology Co., China). The cDNAs were prepared using SuperScript III reverse transcriptase and oligo primers (Nanjing Novizan Biotechnology Co., China). These primers were listed in Table 1.

**Table 1:** Primer sequences used in RT-qPCR analysis.

Name of primer	Sequences
Bax-F	5′-TGGAGCTGCAGAGGATGATT-3′
Bax-R	5′-GGTGTCCAGCCCATGATGGT-3′
Bcl-2-F	5′-GGTGAACTGGGGGAGGATTG-3′
Bcl-2-R	5′-AGCAATCCGACTGTTGAAGTG-3′
cTnT-F	5′-CCAGAGGAGGAGCAACAGAG-3′
cTnT-R	5′-GGCAGGACTTGGGTGTAGAA-3′
LDH-F	5′-ATGGACCTGGATGCTGTGGCT-3′
LDH-R	5′-TCCAGCAGGTGGAGCAGGTT-3′
GAPDH-F	5′-TGATGACATCAAGAAGGTGGTGAAG-3′
GAPDH-R	5′-TCCTTGGAGGCCATGTAGGCCAT-3′
NF-κB-F	5′-CATACGCTGACCCTAGCCTG-3′
NF-κB-R	5′-TCACTGAGCTCCCGATCAGA-3′
HO-1-F	5′-ACACGGGTGACAGAAGAGGCTAA-3′
HO-1-R	5′-CTGTGAGGGACTCTGGTCTTTG-3′
COX-2-F	5′-TTCCAGTATCAGAACCGCATTG-3′
COX-2-R	5′-CAGCAAGTCCGTGTTCAAGGA-3′
Nrf2-F	5′-CTCCGGGAACATAGACAACTTCA-3′
Nrf2-R	5′-TCCTGAGTTTCCATGGTCTGG-3′

### Western blot

H9c2 cells and myocardial tissue were collected, and lysed in ice-cold RIPA lysis buffer. The resulting lysates were mixed with 2× SDS-PAGE sample buffer, boiled for 10 min, and separated by 10% SDS-PAGE. The proteins were then transferred to polyvinylidene difluoride (PVDF) membranes (Millipore, Billerica, MA, USA) and blocked at 37 °C for 60 min with 5% nonfat dry milk. The membranes were incubated with appropriately diluted monoclonal antibodies targeting Bax (#14796, 1:1000, Rabbit, Cell Signaling Technology), COX-2 (#12282, 1:1000, Rabbit, Cell Signaling Technology), NF-κB (#8242, 1:1000, Rabbit, Cell Signaling Technology) and Bcl-2 (#2870, 1:1000, Rabbit, Cell Signaling Technology). After washing, the membranes were treated with horseradish peroxidase-linked goat anti-rabbit IgG secondary antibody (1:5000; Santa Cruz Biotechnology) at 37 °C for 1 h. Protein bands were detected using horseradish peroxidase-conjugated goat anti-rabbit IgG antibodies followed by enhanced chemiluminescence (Pierce Biotechnology, USA).

### EdU cell proliferation experiment

Cells were seeded at 2 × 10⁴ cells/mL concentration in 48-well plates and, cell proliferation was assessed using an EdU kit (C0075S, Beyotime) according to the manufacturer’s instructions.

### Tissue collection, fixation, processing, and sectioning

Mice were deeply anesthetized and euthanized by cervical dislocation. Hearts were rapidly excised, rinsed briefly in cold phosphate-buffered saline to remove residual blood, and transversely sectioned at the papillary muscle level along the short axis. Representative tissue blocks from the left ventricular (LV) free wall and interventricular septum were collected. Samples were fixed in 4% paraformaldehyde for 48 h, processed by routine graded ethanol dehydration and xylene clearing, and paraffin-embedded. Paraffin blocks were serially sectioned using a rotary microtome at a thickness of 4–5 μm. Sections were floated on a 40–45 °C water bath, mounted onto glass slides, and baked at 60 °C for 1 h to enhance tissue adhesion.

### Hematoxylin and eosin (H&E) staining and quantification

Paraffin sections were deparaffinized in xylene and rehydrated through graded ethanol. Sections were stained with hematoxylin (AWI0001a, Abiowell) followed by differentiation and bluing, and counterstained with eosin (AWI0029a, Abiowell). Slides were then dehydrated through graded ethanol, cleared in xylene, and covers lipped with a neutral mounting medium. H&E-stained sections were examined under a bright-field microscope (BA210T, Motic, China). Necrotic areas were identified based on histomorphological features by two independent investigators blinded to group allocation using ImageJ, including disorganized or disrupted myocardial fibers, nuclear pyknosis or loss, increased cytoplasmic eosinophilia, interstitial edema, and inflammatory cell infiltration. The necrotic area and the total myocardial tissue area were manually outlined on the entire section, and the percentage of necrosis was calculated.

### Masson’s trichrome staining and quantification

Paraffin heart sections were deparaffinized and rehydrated as above. Masson’s trichrome staining was performed following the instructions using Masson’s kit (G1340, Solarbio). After staining, slides were dehydrated, cleared in xylene, and coverslipped with a neutral mounting medium. Images were acquired using a bright-field microscope (BA210T, Motic, China). Collagen deposition (blue-stained area) was quantified using ImageJ by threshold-based segmentation, and expressed as collagen area fraction (%) = collagen-positive area / total myocardial area (excluding the ventricular cavity). For each animal, multiple non-overlapping fields across ≥ 3 sections were analyzed and averaged.

### Sirius red staining and quantification

Paraffin-embedded heart sections were deparaffinized in xylene and rehydrated through graded ethanol. Sections were stained with Sirius Red solution according to the manufacturer’s protocol (C0190M, Beyotime), rinsed, briefly differentiated in acidified water, and then dehydrated through graded ethanol, cleared in xylene, and covers lipped with a neutral mounting medium. Stained sections were imaged using a bright-field microscope equipped with a polarized-light module (BA210T, Motic, China), and images were captured under both bright-field and polarized-light conditions.

For quantitative analysis, ImageJ was used to calculate the area fraction of Sirius Red–positive signals. Under bright-field imaging, the yellow-colored Sirius Red–positive area was segmented using a consistent color threshold and expressed as yellow area / total tissue area (%) within each field of view. Under polarized light, birefringent collagen signals were separated and quantified as green area / total tissue area (%) and yellow area / total tissue area (%), respectively, using identical threshold settings across groups. For each animal, multiple non-overlapping fields from predefined regions were analyzed across ≥ 3 sections, and the mean value was used for statistical comparisons. All quantification was performed by investigators blinded to group allocation.

### Statistical analysis

GraphPad prism 10.0 software and ImageJ software (version 1.54) were used for data processing. Data were stated as mean ± SD. Two group comparisons were done using t test, and multiple comparisons were conducted with one-way ANOVA analysis using Bonferroni’s multiple comparisons test. Difference was considered to be significant if *P* < 0.05 (*p < 0.05, **p < 0.01, ***p < 0.001, ****p < 0.0001, “ns” indicates no significant).

## Results

### Isolation and characterization of COPD-associated extracellular vesicles

**Fig. 1:**
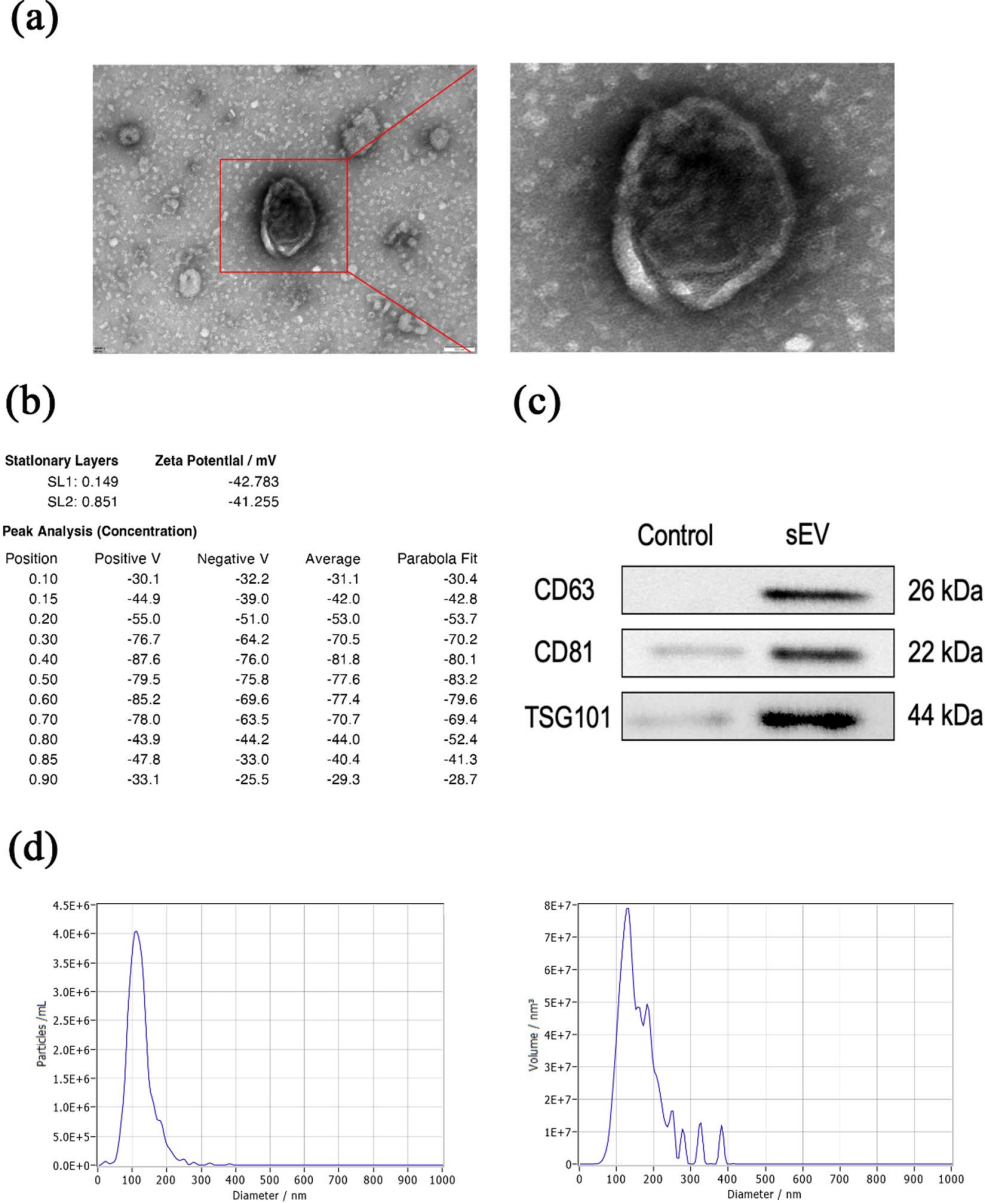
Isolation and characterization of extracellular vesicles. (**a**) Transmission electron microscopy (TEM) image of the isolated extracellular vesicles. (**b**) Zeta potential measurement of extracellular vesicles, indicating their surface charge, which reflects the stability of the vesicles. (**c**) Western blot analysis demonstrating the presence of sEV-specific markers, CD63, CD81, and TSG101. (**d**) Nanoparticle tracking analysis (NTA) showing the size distribution and concentration of the extracellular vesicles.

The successful isolation and identification of extracellular vesicles forms a crucial foundation for subsequent functional studies. This study systematically characterized extracellular vesicles derived from COPD-associated cells. Transmission electron microscopy (TEM) observations revealed that the isolated vesicles exhibited typical cup-shaped or circular structures with distinct double-membrane features, maintaining morphological integrity and uniform distribution, consistent with the ultrastructural characteristics of extracellular vesicles (Fig. 1a). Further Zeta potential analysis revealed an sEVs surface potential of approximately − 42.783 mV, indicating excellent dispersion and high stability in solution. This property facilitates subsequent experimental procedures and in vitro/in vivo applications (Fig. 1b).

To further validate the molecular characteristics of extracellular vesicles, WB analysis was performed to detect sEVs-specific marker proteins. Results showed that CD63, CD81, and TSG101 exhibited distinct positive expression in the sEVs samples, while no significant bands were observed in the control group (untreated HUVEC cells). This indicates that the isolated vesicles possess typical sEVs protein features and high purity (Fig. 1c). Additionally, nanoparticle tracking analysis (NTA) revealed that the sEVs particle size predominantly ranged from 80 to 150 nm with a concentrated distribution, highly consistent with the size characteristics of classical extracellular vesicles (Fig. 1d).

In summary, the aforementioned multifaceted characterization results confirm the successful isolation and high-purity preparation of COPD-associated extracellular vesicles at multiple levels, including morphology, physicochemical properties, and molecular markers. This establishes a reliable foundation for subsequent investigations into their biological functions in myocardial injury.

### Effects of COPD-associated extracellular vesicles on cardiomyocyte injury

**Fig. 2:**
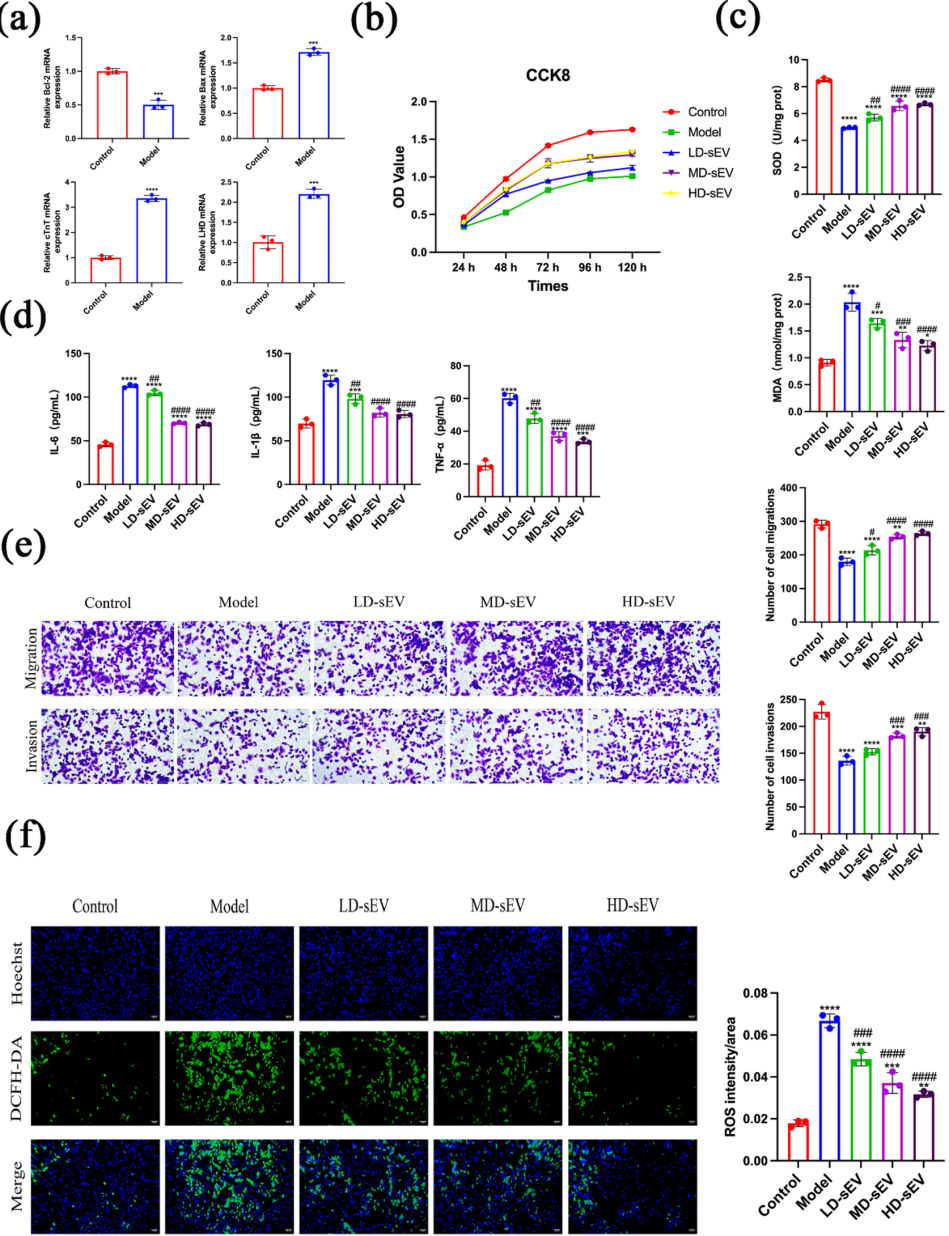
Effects of COPD-related extracellular vesicles on myocardial cell injury. (**a**) RT-qPCR analysis of Bax, cTnT, and Bcl-2 mRNA expression levels in myocardial cells after treatment with different doses of extracellular vesicles. (**b**) CCK-8 assay results showing cell viability in the model group and after treatment with extracellular vesicles at different doses. (**c**) Measurement of SOD activity and MDA content in the model group and after treatment with different doses of extracellular vesicles. (**d**) ELISA analysis of IL-6, TNF-α, and IL-1β levels in the model group and after treatment with extracellular vesicles at different doses, indicating the inflammatory response. (**e**) Transwell assay results assessing cell migration in the model group and after treatment with different doses of extracellular vesicles. (f) Left: DCFH-DA fluorescence staining results showing oxidative stress levels in the model group and after treatment with different doses of extracellular vesicles. Right: ROS intensity quantification. *Indicates comparison with Control, # indicates comparison with Model group. *n* = 3. Data are presented as mean ± SD. Statistical analysis: one-way ANOVA with Bonferroni’s multiple comparisons test. Significance: *p < 0.05, **p < 0.01, ***p < 0.001, ****p < 0.0001; ns, not significant.

Apoptosis, oxidative stress, and inflammatory responses are widely regarded as key pathological processes driving the onset and progression of cardiomyocyte injury. The Bcl-2 family plays a central role in regulating mitochondria-dependent apoptosis, in which Bax upregulation and Bcl-2 downregulation are commonly indicative of apoptotic activation^18^. RT-qPCR analysis showed that, compared with the control group, the injury model exhibited significantly increased expression of pro-apoptotic and myocardial injury–related genes, including Bax and cTnT, accompanied by a marked decrease in the anti-apoptotic gene Bcl-2, confirming successful establishment of the cardiomyocyte injury model (Fig. 2a). Consistently, CCK-8 assays demonstrated a pronounced reduction in cell viability in the model group, whereas treatment with COPD-associated extracellular vesicles restored cell viability in a dose-dependent manner, suggesting that extracellular vesicles effectively attenuate cardiomyocyte injury (Fig. 2b).

Oxidative stress is another critical contributor to cardiomyocyte injury and is typically characterized by reduced antioxidant enzyme activity and increased accumulation of lipid peroxidation products^19^. In the study, superoxide dismutase (SOD) activity was significantly decreased, while malondialdehyde (MDA) levels were markedly increased in the model group. sEVs intervention substantially restored SOD activity and reduced MDA levels, indicating alleviation of oxidative stress–mediated damage **(**Fig. 2c**)**. In addition, ELISA results revealed significantly elevated levels of the pro-inflammatory cytokines IL-6, TNF-α, and IL-1β in the model group, all of which were markedly reduced following sEVs treatment, supporting a robust anti-inflammatory effect **(**Fig. 2d**)**.

Impaired migratory and invasive capacities represent important features of functional deterioration after cardiomyocyte injury and can directly influence subsequent repair and remodeling. Transwell assays showed that migration and invasion were substantially diminished in the model group compared with controls, whereas sEVs treatment markedly improved both capacities, indicating functional recovery under injurious conditions **(**Fig. 2e**)**. Furthermore, DCFH-DA fluorescence staining demonstrated a significant increase in intracellular ROS production in the model group, which was effectively suppressed by sEVs treatment, further supporting that extracellular vesicles mitigate cellular injury through attenuation of oxidative stress **(**Fig. 2f**)**. These findings indicate that COPD-associated extracellular vesicles confer cytoprotection against cardiomyocyte injury by suppressing apoptosis, reducing oxidative stress, and dampening inflammatory responses.

### COPD-associated extracellular vesicles improve mitochondrial function and suppress apoptosis in cardiomyocytes

**Fig. 3:**
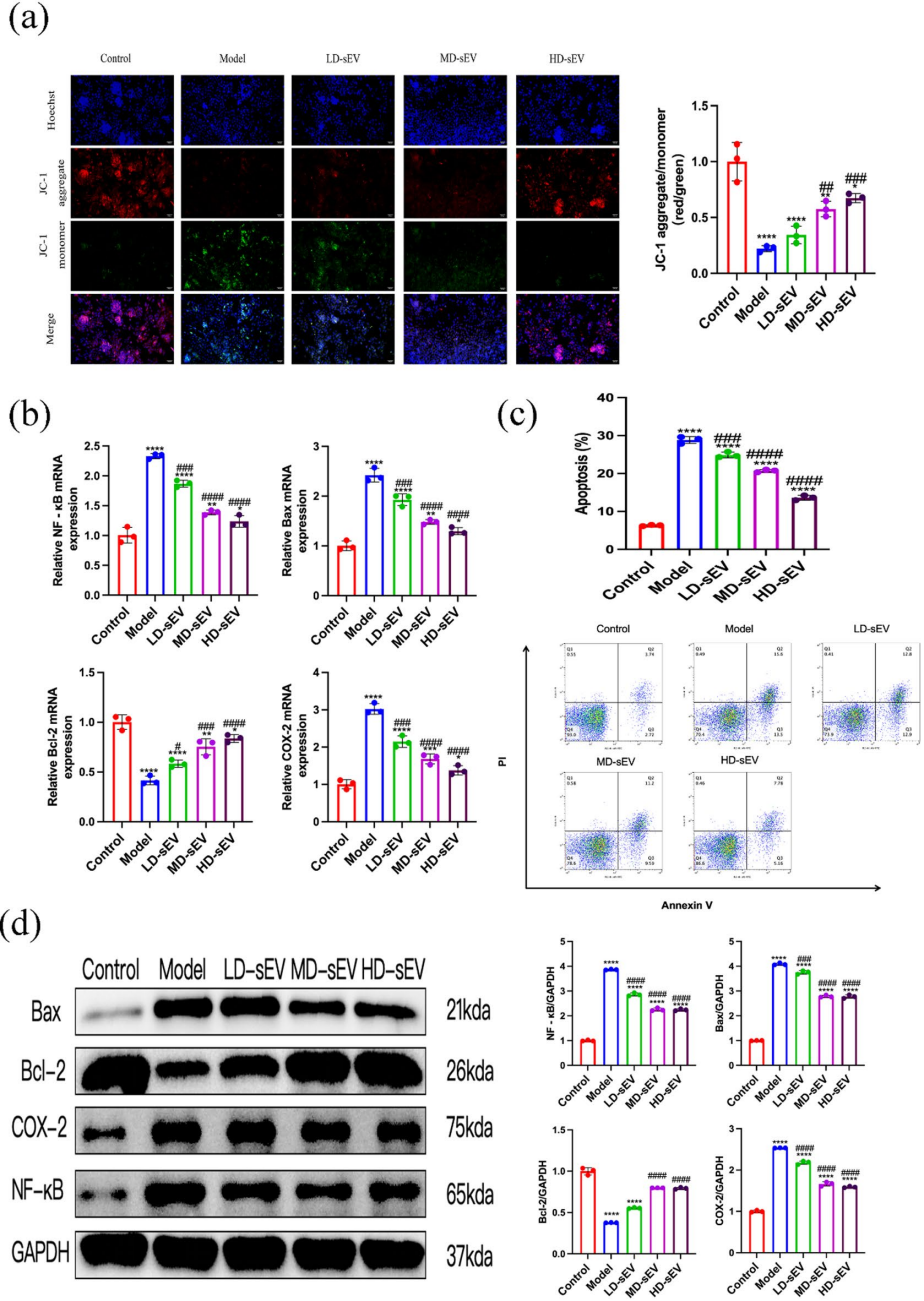
COPD-related extracellular vesicles improve mitochondrial function and inhibit apoptosis in myocardial cells. (**a**) Left: JC-1 staining results showing mitochondrial membrane potential in the model group and after sEVs treatment. Right: JC-1 aggregate/monomer quantification. (**b**) RT-qPCR analysis of Bax, NF-κB, COX-2, and Bcl-2 mRNA expression levels in the model group and after sEVs treatment.(**c**) Apoptosis analysis in the model group and after sEVs treatment, demonstrating the impact of extracellular vesicles on cell apoptosis. (**d**) Western blot analysis of Bax, NF-κB, COX-2, and Bcl-2 protein expression levels in the model group and after sEVs treatment. *Indicates comparison with Control, # indicates comparison with Model group. *n* = 3. Data are presented as mean ± SD. Statistical analysis: one-way ANOVA with Bonferroni’s multiple comparisons test. Significance: *p < 0.05, **p < 0.01, ***p < 0.001, ****p < 0.0001; ns, not significant.

Mitochondrial dysfunction and mitochondria-mediated apoptosis constitute critical pathological bases of cardiomyocyte injury. A decline in mitochondrial membrane potential (ΔΨm) typically reflects mitochondrial depolarization and activation of the intrinsic apoptotic pathway. JC-1 staining showed that, compared with the control group, cardiomyocytes in the injury model exhibited a marked reduction in ΔΨm, as evidenced by a significantly decreased red/green fluorescence ratio, indicating pronounced mitochondrial depolarization. Notably, treatment with COPD-associated extracellular vesicles at increasing doses led to a progressive enhancement of red fluorescence and a restoration of ΔΨm, suggesting that extracellular vesicles effectively preserve mitochondrial function **(**Fig. 3a**)**.

We next assessed the expression of genes involved in mitochondrial apoptosis and inflammation by RT–qPCR. The model group displayed significant upregulation of the pro-apoptotic gene Bax and the stress/inflammation-related genes NF-κB and COX-2, together with a marked downregulation of the anti-apoptotic gene Bcl-2. These aberrant expression patterns were partially reversed following sEVs treatment (Fig. 3b). Consistently, flow cytometric analysis demonstrated a significantly increased apoptotic rate in the model group, whereas sEVs administration markedly reduced apoptosis in a dose-dependent manner (Fig. 3c).

Western blotting further corroborated these molecular changes. Compared with the model group, sEVs treatment significantly decreased protein levels of Bax, NF-κB, and COX-2, while increasing Bcl-2 expression. Collectively, these data indicate that COPD-associated extracellular vesicles protect cardiomyocytes by improving mitochondrial function, suppressing the mitochondria-dependent apoptotic pathway, and attenuating inflammatory signaling (Fig. 3d).

### COPD-associated extracellular vesicles improve cardiac function in mice after myocardial infarction

**Fig. 4:**
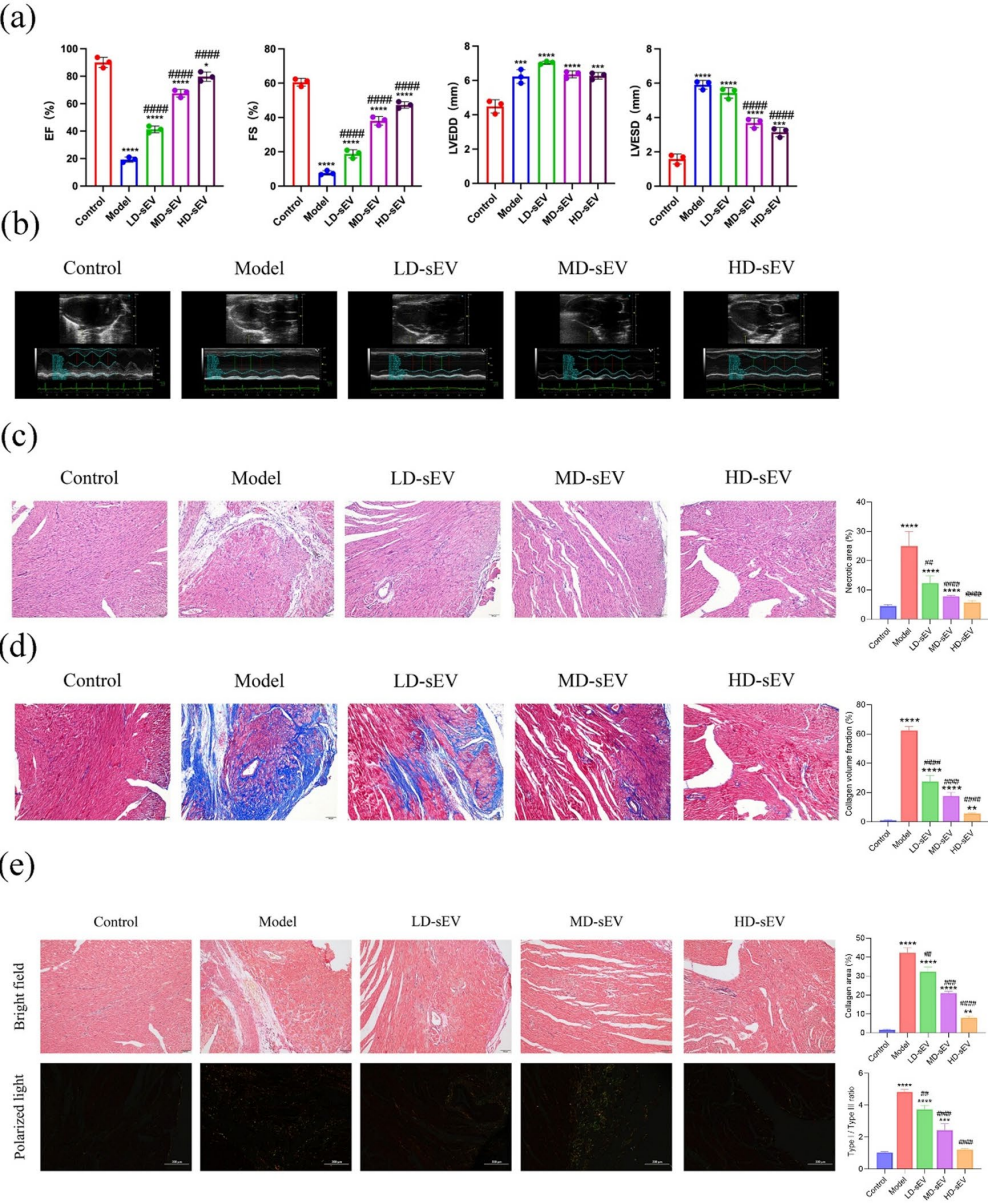
COPD-related extracellular vesicles improve cardiac function in myocardial infarction mice. (**a**) Echocardiography results showing the EF, fractional shortening FS, LVEDD, and LVESD in the model group and after sEVs treatment. (**b**) Cardiac ultrasound imaging of mice in the model group and after sEVs treatment, illustrating the structural changes in the heart. (**c**) Hematoxylin and eosin (HE) staining results showing histological changes in the hearts of the model group and after sEVs treatment. (**d**) Masson’s trichrome staining results demonstrating the extent of fibrosis in the hearts of the model group and after sEVs treatment. (**e**) Sirius Red staining results showing collagen deposition and fibrosis in the hearts of the model group and after sEVs treatment. *Indicates comparison with Control, # indicates comparison with Model group. *n* = 3. Data are presented as mean ± SD. Statistical analysis: one-way ANOVA with Bonferroni’s multiple comparisons test. Significance: *p < 0.05, **p < 0.01, ***p < 0.001, ****p < 0.0001; ns, not significant.

Impaired cardiac function and adverse ventricular remodeling are major pathological features underlying heart failure development following myocardial infarction. Echocardiographic assessment showed that, compared with the sham-operated group, MI mice exhibited significantly reduced ejection fraction (EF) and FS, accompanied by increased LVEDD and LVESD, indicating impaired systolic function and evident ventricular dilation. Administration of COPD-associated extracellular vesicles at increasing doses progressively restored EF and FS and significantly improved LVEDD and LVESD, demonstrating a marked improvement in post-infarction cardiac function **(**Fig. 4a**)**.

These functional benefits were further supported by representative echocardiographic images, which showed enhanced myocardial contractility in the sEVs-treated groups (Fig. 4b). Histological examination by H&E staining revealed disrupted myocardial fibers, disorganized architecture, and extensive necrosis in MI hearts, whereas sEVs intervention gradually improved tissue integrity and preserved myocardial structure (Fig. 4c). In parallel, Masson’s trichrome staining demonstrated pronounced collagen deposition and aggravated fibrosis in the MI group, which was substantially reduced following sEVs treatment (Fig. 4d). Sirius Red staining further confirmed robust collagen accumulation in the MI myocardium and showed that sEVs administration markedly decreased collagen content and improved collagen distribution patterns (Fig. 4e). Taken together, these results indicate that COPD-associated extracellular vesicles significantly improve cardiac function after MI, inhibit myocardial fibrosis, and mitigate adverse ventricular remodeling, thereby exerting robust cardioprotective effects in vivo.

### COPD-associated extracellular vesicles suppress inflammatory responses and apoptotic signaling in infarcted myocardium

**Fig. 5:**
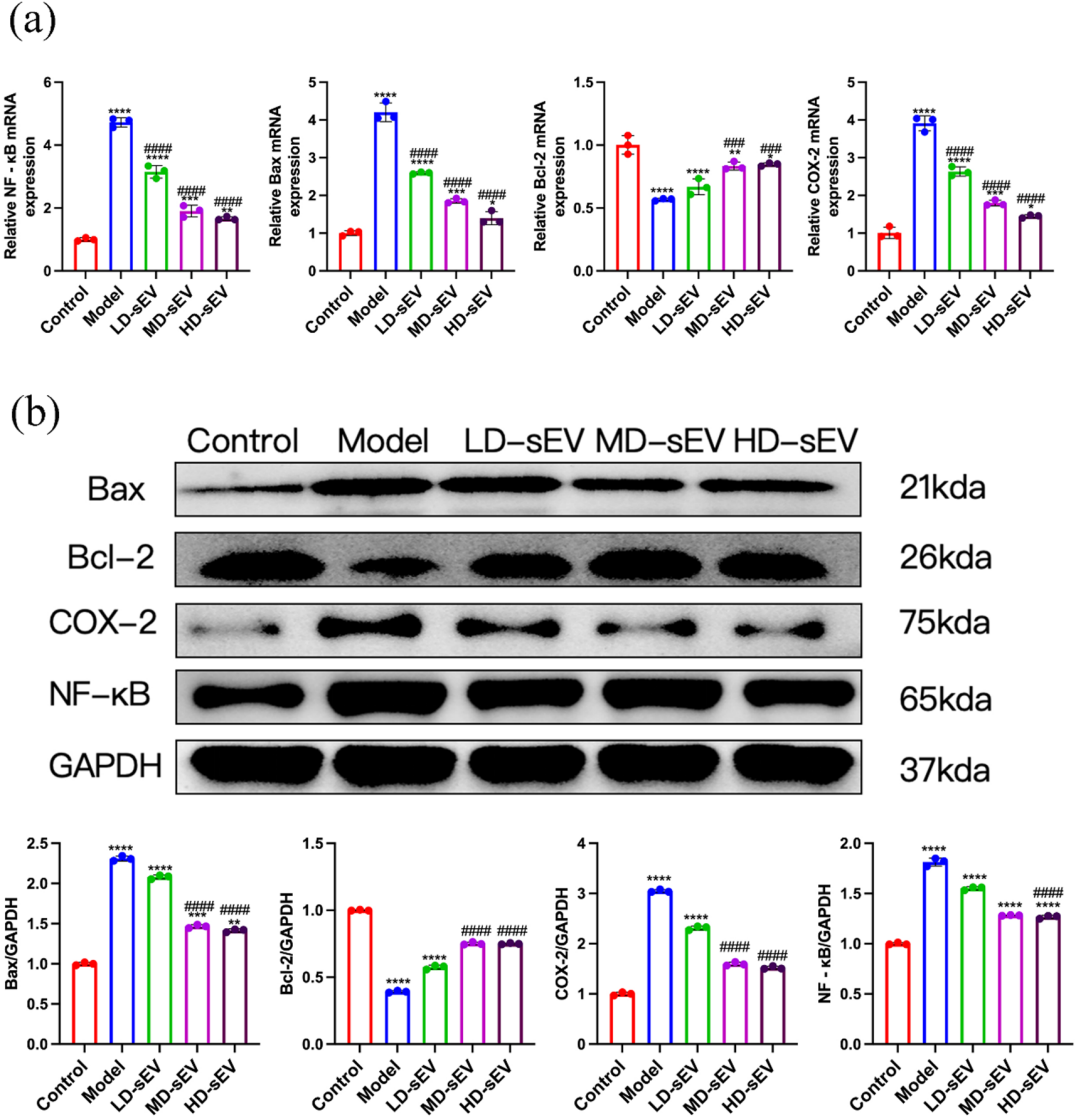
COPD-related extracellular vesicles inhibit inflammatory response and apoptotic pathways in myocardial tissue. (**a**) RT-qPCR analysis showing the mRNA expression levels of NF-κB, Bax, COX-2, and Bcl-2 in myocardial tissue after sEVs treatment compared to the model group. (**b**) Western blot analysis further validating the protein expression levels of NF-κB, Bax, COX-2, and Bcl-2. *Indicates comparison with Control, # indicates comparison with Model group. *n* = 3. Data are presented as mean ± SD. Statistical analysis: one-way ANOVA with Bonferroni’s multiple comparisons test. Significance: *p < 0.05, **p < 0.01, ***p < 0.001, ****p < 0.0001; ns, not significant.

Sustained inflammatory activation and inflammation-associated apoptosis play pivotal roles in myocardial injury and adverse remodeling after myocardial infarction, with the NF-κB signaling pathway serving as a key molecular hub regulating pro-inflammatory cytokine production and apoptosis-related gene expression^20^. RT–qPCR analysis showed that, compared with the sham group, myocardial tissues from MI mice exhibited significantly increased expression of the pro-inflammatory and pro-apoptotic genes NF-κB, Bax, and COX-2, whereas expression of the anti-apoptotic gene Bcl-2 was markedly decreased. Treatment with COPD-associated extracellular vesicles at different doses significantly mitigated these abnormalities, as reflected by reduced expression of NF-κB, Bax, and COX-2 and increased Bcl-2 expression (Fig. 5a). Western blotting further confirmed the transcriptional findings at the protein level. Compared with the MI model group, sEVs treatment markedly suppressed protein expression of NF-κB, Bax, and COX-2, while significantly upregulating Bcl-2 (Fig. 5b). These results indicate that COPD-associated extracellular vesicles confer cardioprotection by inhibiting NF-κB–mediated inflammatory signaling and restoring the Bax/Bcl-2 balance, thereby alleviating myocardial inflammation and apoptosis.

### COPD-associated extracellular vesicles attenuate cardiomyocyte injury via activation of the PI3K/Akt pathway

**Fig. 6:**
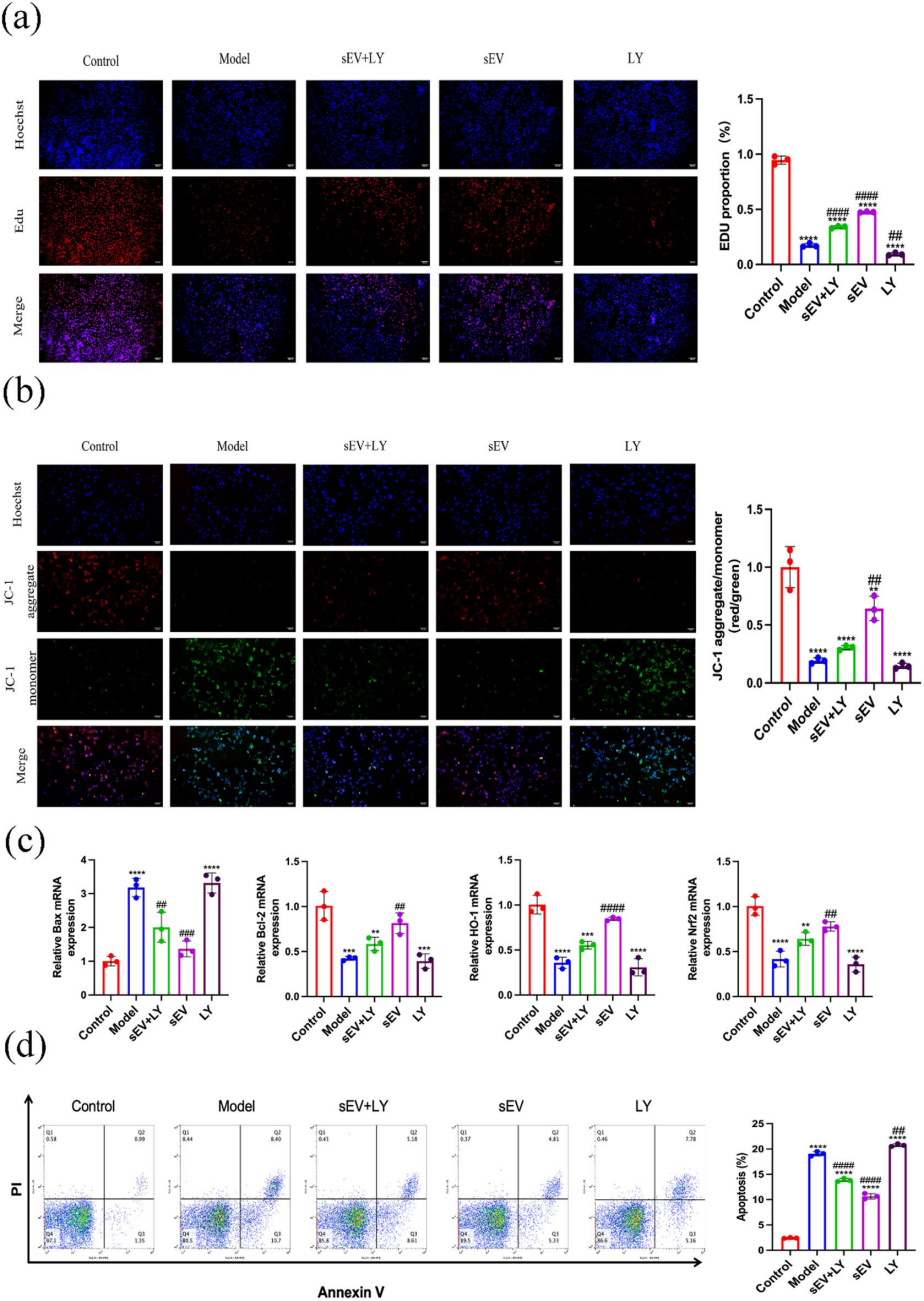
COPD-related extracellular vesicles improve myocardial cell injury via activation of the PI3K/Akt pathway. (**a**) EdU staining results showing cell proliferation in the Control, Model, sEV + LY, sEV, and LY groups. (**b**) JC-1 staining results demonstrating mitochondrial membrane potential in the Control, Model, sEV + LY, sEV, and LY groups. (**c**) RT-qPCR analysis of Bax, Bcl-2, Nrf2, and HO-1 mRNA expression levels in the Control, Model, sEV + LY, sEV, and LY groups. (**d**) Flow cytometry analysis of apoptosis rates in the Control, Model, sEV + LY, sEV, and LY groups. LY: LY294002. * indicates comparison with Control, # indicates comparison with Model group. *n* = 3. Data are presented as mean ± SD. Statistical analysis: one-way ANOVA with Bonferroni’s multiple comparisons test. Significance: *p < 0.05, **p < 0.01, ***p < 0.001, ****p < 0.0001; ns, not significant.

The PI3K/Akt signaling pathway plays a critical role in regulating cardiomyocyte survival, proliferation, and anti-apoptotic and antioxidant responses^21^. To further elucidate the molecular mechanism by which COPD-associated extracellular vesicles alleviate cardiomyocyte injury, we performed pathway blockade experiments using the PI3K/Akt-specific inhibitor LY294002. Cells were assigned to five groups: Control, Model, sEVs + LY, LY, and sEVs (sEVs, High Dose, 100 µg/mL). LY294002 was applied at a final concentration of 10 µM for 1 h prior to sEVs treatment and maintained throughout the reoxygenation period. Extracellular vesicles were administered at 100 µg/mL at the onset of reoxygenation. EdU staining showed that cardiomyocyte proliferative capacity was markedly reduced in the model group compared with controls; sEVs treatment substantially restored proliferation, whereas co-treatment with LY294002 significantly weakened the pro-proliferative effect, indicating that sEVs-mediated enhancement of cardiomyocyte proliferation depends on PI3K/Akt activation (Fig. 6a).

In parallel, JC-1 staining demonstrated a pronounced reduction in mitochondrial membrane potential in the model group. sEVs administration significantly increased the red/green fluorescence ratio, consistent with improved mitochondrial function; however, this restorative effect on mitochondrial membrane potential was partially offset when PI3K/Akt signaling was inhibited by LY294002 (Fig. 6b). RT–qPCR analysis further revealed that sEVs treatment significantly downregulated the pro-apoptotic gene Bax, while upregulating Bcl-2 and the antioxidant-associated factors Nrf2 and HO-1; these protective transcriptional changes were attenuated upon LY294002 co-treatment (Fig. 6c). Consistently, flow cytometry showed that sEVs treatment markedly reduced cardiomyocyte apoptosis, whereas apoptosis was significantly increased when PI3K/Akt signaling was pharmacologically inhibited (Fig. 6d). These findings suggested that PI3K/Akt activation served as a functional component of the EV-mediated protective response against cardiomyocyte injury. However, the partial reversal by LY294002 indicates that PI3K/Akt signaling contributes to, but may not fully account for, the observed effects.

## Discussion

Although chronic obstructive pulmonary disease (COPD) is associated with an increased risk of myocardial infarction (MI) and poorer post-infarction outcomes, with systemic inflammation and oxidative stress being well-recognized as key drivers of impaired cardiac repair in this comorbid setting, accumulating evidence has revealed that sEVs derived from COPD-associated cells exert a protective effect against myocardial injury and remodeling rather than exacerbating these pathological processes^17,22^. Using complementary in vitro and in vivo models, we demonstrated that COPD-associated sEVs attenuated ischemic myocardial injury phenotypes and improve post-MI cardiac function, and we provided functional evidence that PI3K/Akt signaling at least in part contributes to these effects.

Across the cardiomyocyte injury assays, sEVs treatment produced a coherent protective profile. Extracellular vesicles increased cell viability in a dose-dependent manner and reduced oxidative stress. Extracellular vesicles also suppressed the elevation of pro-inflammatory cytokines, improved migration/invasion capacity, and reduced apoptosis. These cellular effects were accompanied by a shift in key stress-response and apoptosis-associated molecules, including reduced Bax and increased Bcl-2. Mitochondrial dysfunction is a central determinant of cardiomyocyte death after ischemic stress, and loss of mitochondrial membrane potential is closely linked to intrinsic apoptosis through Bcl-2 family regulation^23,24^. Consistently, JC-1 analyses showed that extracellular vesicles restored mitochondrial membrane potential, supporting preservation of mitochondrial integrity as part of the cytoprotective phenotype.

The in vitro findings were paralleled by organ-level benefits after MI. sEVs administration improved systolic function and attenuated ventricular dilation, indicating mitigation of adverse remodeling. Histological analyses further showed reduced myocardial injury and collagen deposition. Adverse post-MI remodeling is driven by early cardiomyocyte loss and sustained inflammatory activation that promote fibrotic replacement and chamber dilation^25,26^. The concordant improvements in apoptosis- and inflammation-associated readouts, together with reduced fibrosis, support the interpretation that extracellular vesicles modulate upstream injury processes that shape later remodeling trajectories. Nonetheless, additional time-resolved analyses will be required to define the temporal window in which extracellular vesicles exert their dominant effects.

PI3K/Akt signaling contributes substantially to sEVs-mediated protection. The PI3K/Akt pathway is a well-established regulator of cardiomyocyte survival, proliferation, and stress adaptation, with documented roles in limiting ischemia-induced apoptosis and supporting mitochondrial homeostasis^27,28^. In our study, pharmacologic inhibition of PI3K/Akt with LY294002 weakened multiple sEVs-associated benefits. The Nrf2/HO-1 axis represents a major cellular antioxidant program implicated in protection against ischemic and oxidative injury, and PI3K/Akt has been linked to Nrf2 activation in several cell contexts^29^. In infarcted myocardium, sEVs treatment reduced NF-κB and COX-2 expression while restoring the Bax/Bcl-2 balance, consistent with attenuation of inflammation-associated apoptotic signaling. NF-κB is widely recognized as a central transcriptional regulator of post-ischemic inflammatory responses and can amplify myocardial injury by promoting cytokine production and downstream stress pathways^30^. Given the bidirectional coupling between oxidative stress and NF-κB activation, reductions in ROS and inflammatory gene expression observed here likely reinforce each other to shift the post-injury milieu toward resolution rather than propagation of damage.

Despite the observed involvement of PI3K/Akt signaling, several limitations of the present mechanistic study must be acknowledged. First, although the PI3K/Akt inhibitor LY294002 significantly attenuated the cardioprotective effects of COPD-associated sEVs, this reversal was only partial. Given that PI3K/Akt signaling is both ubiquitous and pleiotropic, and that LY294002 may exert potential off-target effects, these findings should be interpreted with caution. Furthermore, while we demonstrated functional dependency using a pharmacologic approach, direct biochemical evidence—such as the quantification of Akt phosphorylation (p-Akt) or the activation of downstream targets (e.g., mTOR, GSK3β, or FoxO)—was not provided in the current work. Together, these data suggested that PI3K/Akt signaling contributes to, but likely does not fully account for, the complex integrated effects elicited by COPD-associated sEVs.

A key limitation of this study is the inherent heterogeneity of EVs whose cargo composition and biological effects are strongly influenced by donor cell type and disease context. In the present work, the COPD-mimetic sEVs were derived from CSE-stimulated bronchial epithelial cells, and therefore our conclusions should be interpreted as specific to this epithelial EV subtype. EVs released from other COPD-relevant sources, particularly inflammatory cells such as macrophages, as well as circulating EVs isolated from patients, may carry distinct molecular signatures and could exert different, and in many settings pro-inflammatory, effects. Accordingly, future studies should (i) perform head-to-head comparisons of EVs derived from multiple COPD-associated donor cell types under matched exposure conditions, (ii) define the key bioactive cargo responsible for cardioprotective versus injurious phenotypes, and (iii) validate the present findings using patient-derived circulating sEVs across clinically relevant states. Such work will be essential to delineate the boundaries of applicability and to establish whether COPD-associated EVs can be leveraged for therapeutic modulation in ischemic heart disease.

Collectively, this study supports that COPD-associated extracellular vesicles attenuate cardiomyocyte injury and improve post-MI functional and structural outcomes, with PI3K/Akt signaling contributing to mitochondrial preservation, antioxidant responses, and suppression of apoptosis and inflammatory signaling. PI3K/Akt signaling contributes to, but may not fully account for, the observed effects. These findings provide a mechanistic basis for further exploration of sEVs-based interventions for ischemic heart disease, particularly in the setting of cardiopulmonary comorbidity.

## Supplementary Information

Below is the link to the electronic supplementary material.

Supplementary Material 1

## Data Availability

All data generated or analyzed during this study are included in this article and its online supplementary material. Further inquiries can be directed to the corresponding author.
