# Supplementary figures and images for: Targeted therapies for myocardial infarction based on COPD-related extracellular vesicles
Source: Sci Rep. 2026 May 11;16:21479. doi: 10.1038/s41598-026-50402-8 (PMC13350939; doi:10.1038/s41598-026-50402-8)

Bax-cell


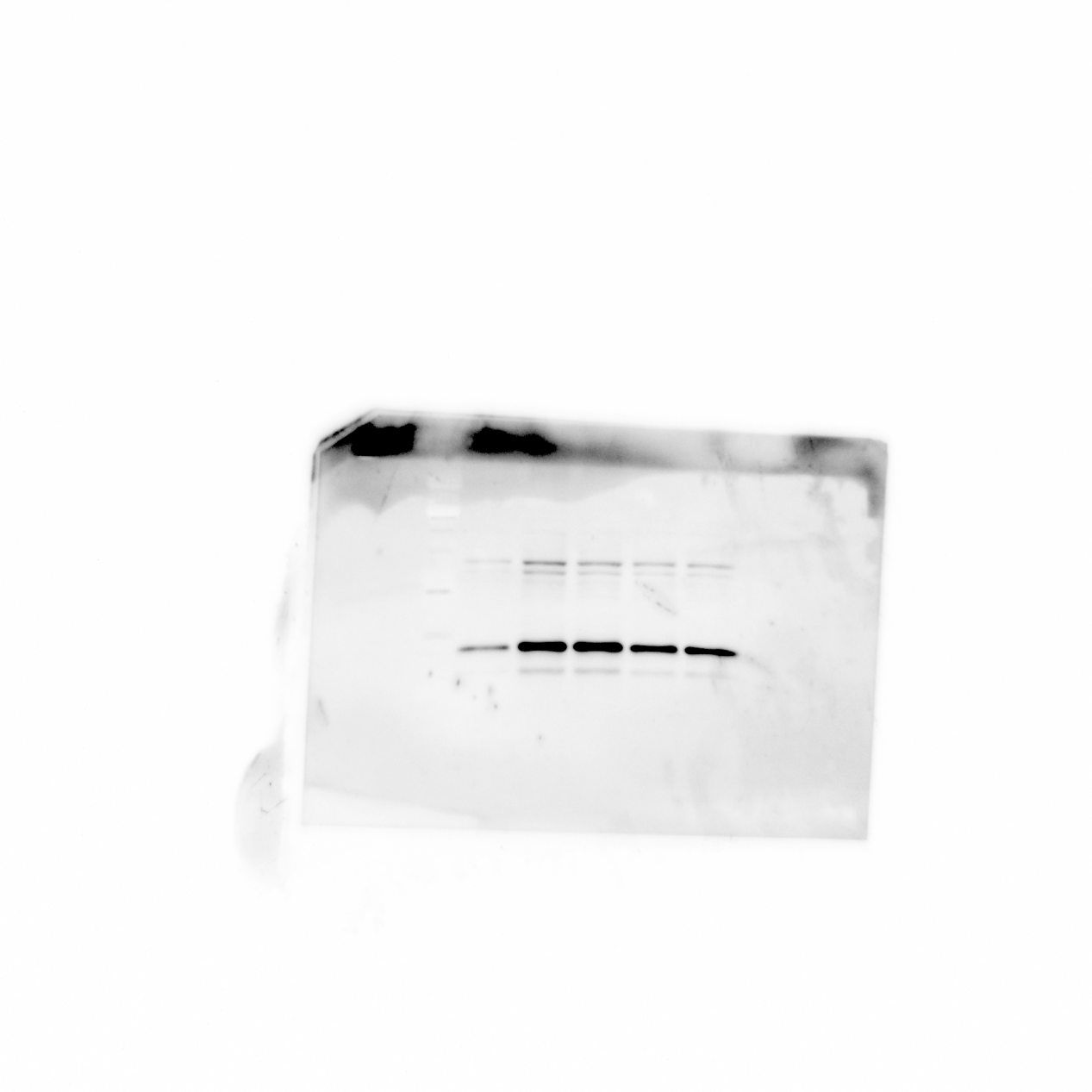


Bcl-2-cell


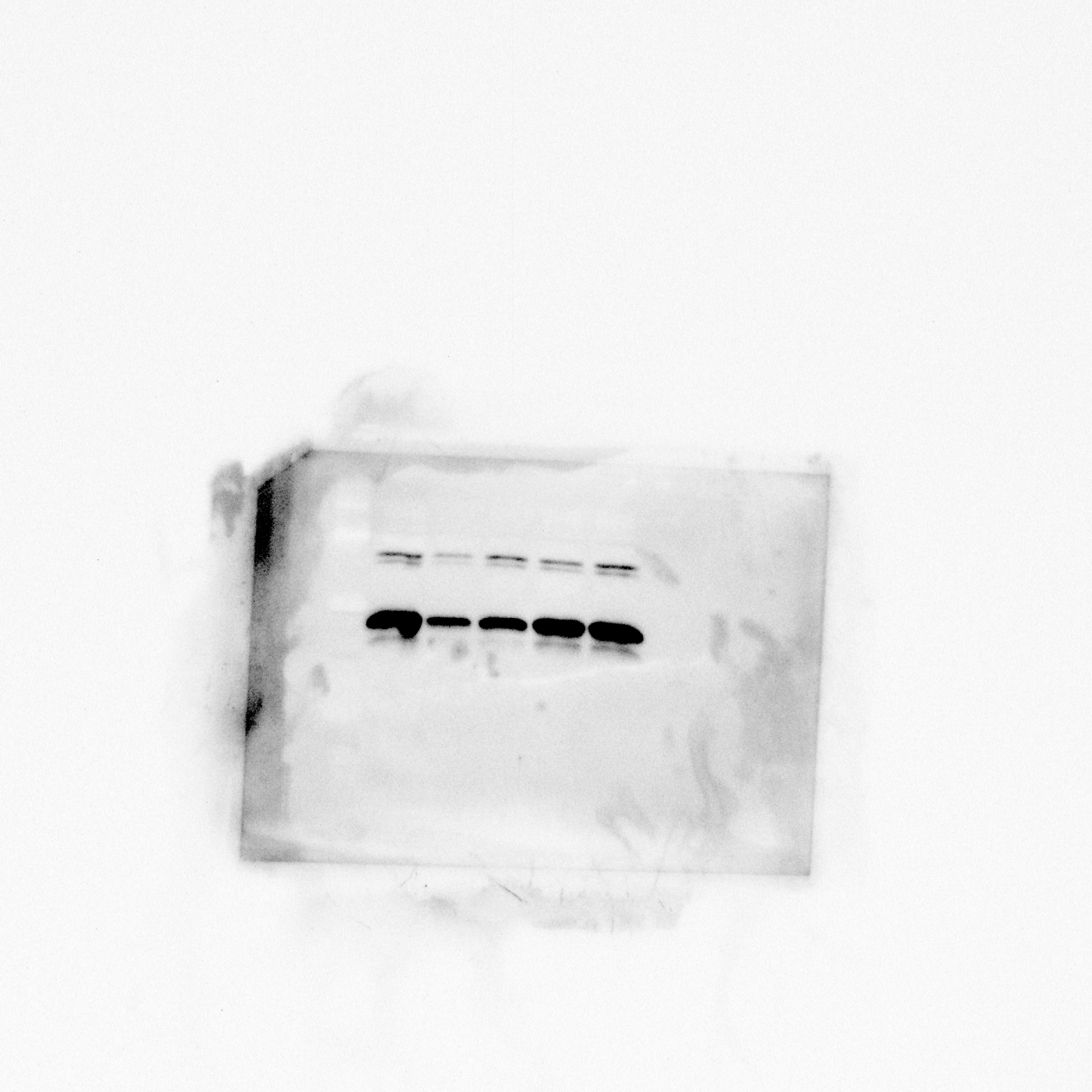


COX2-cell


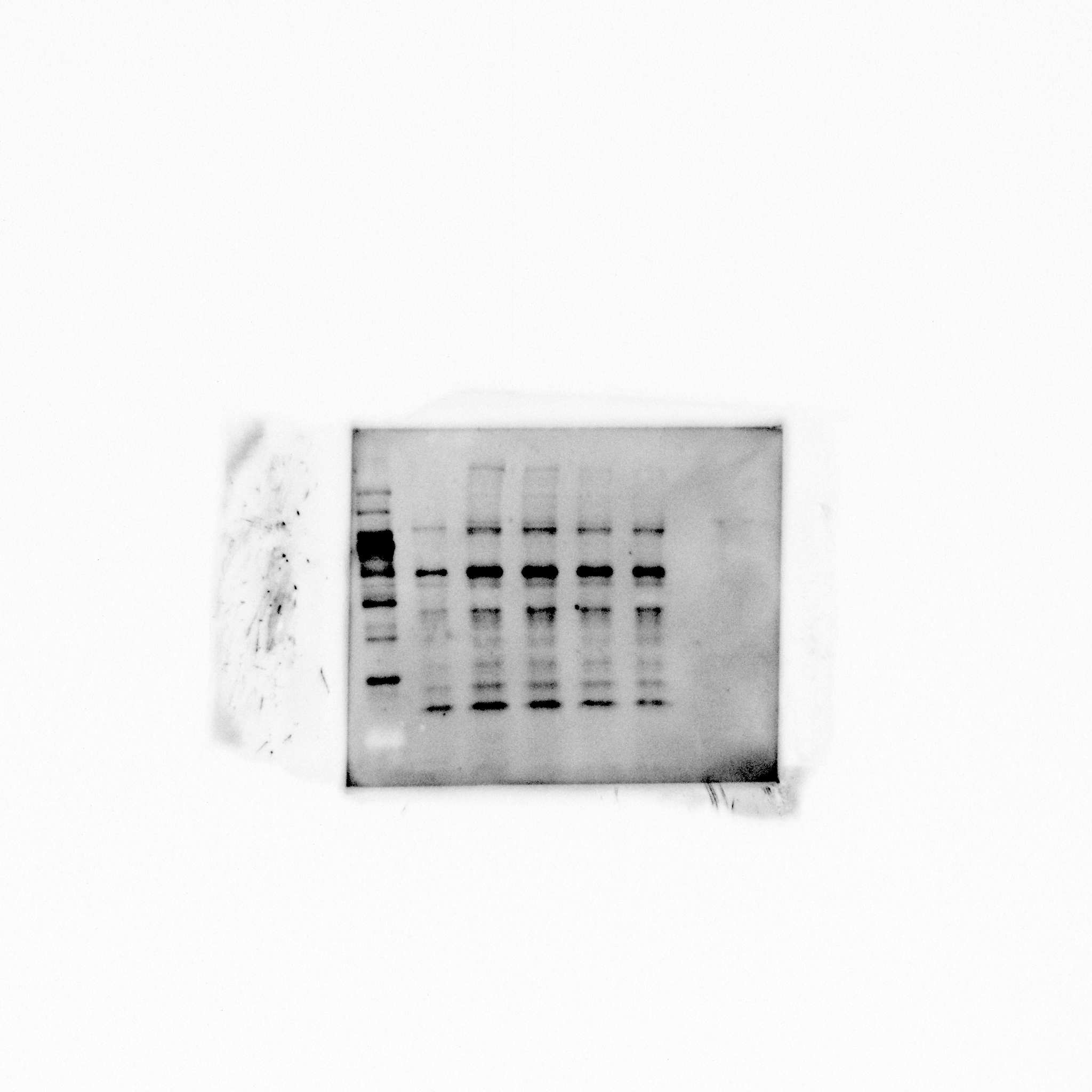


NF-kB-cell


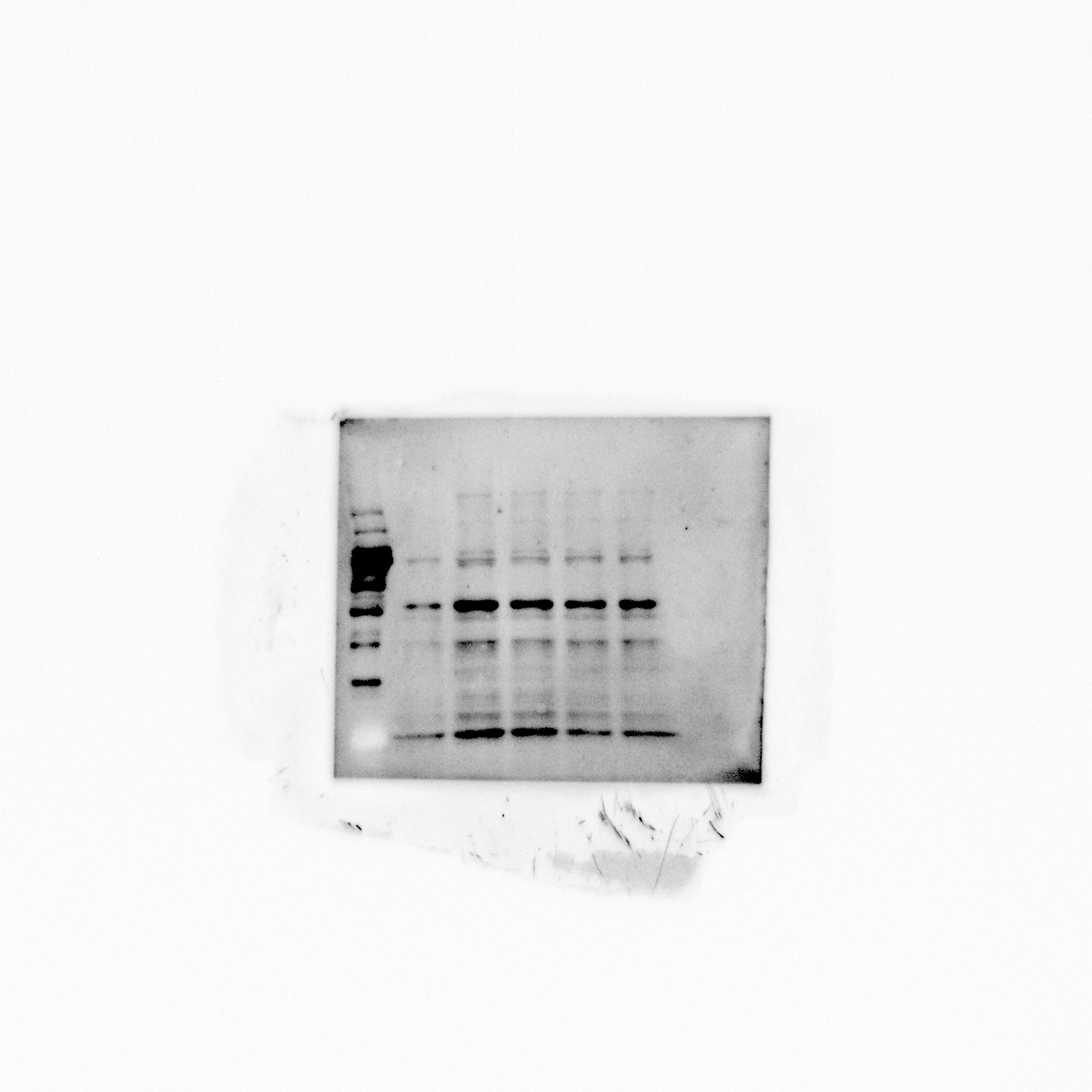


GAPDH-cell


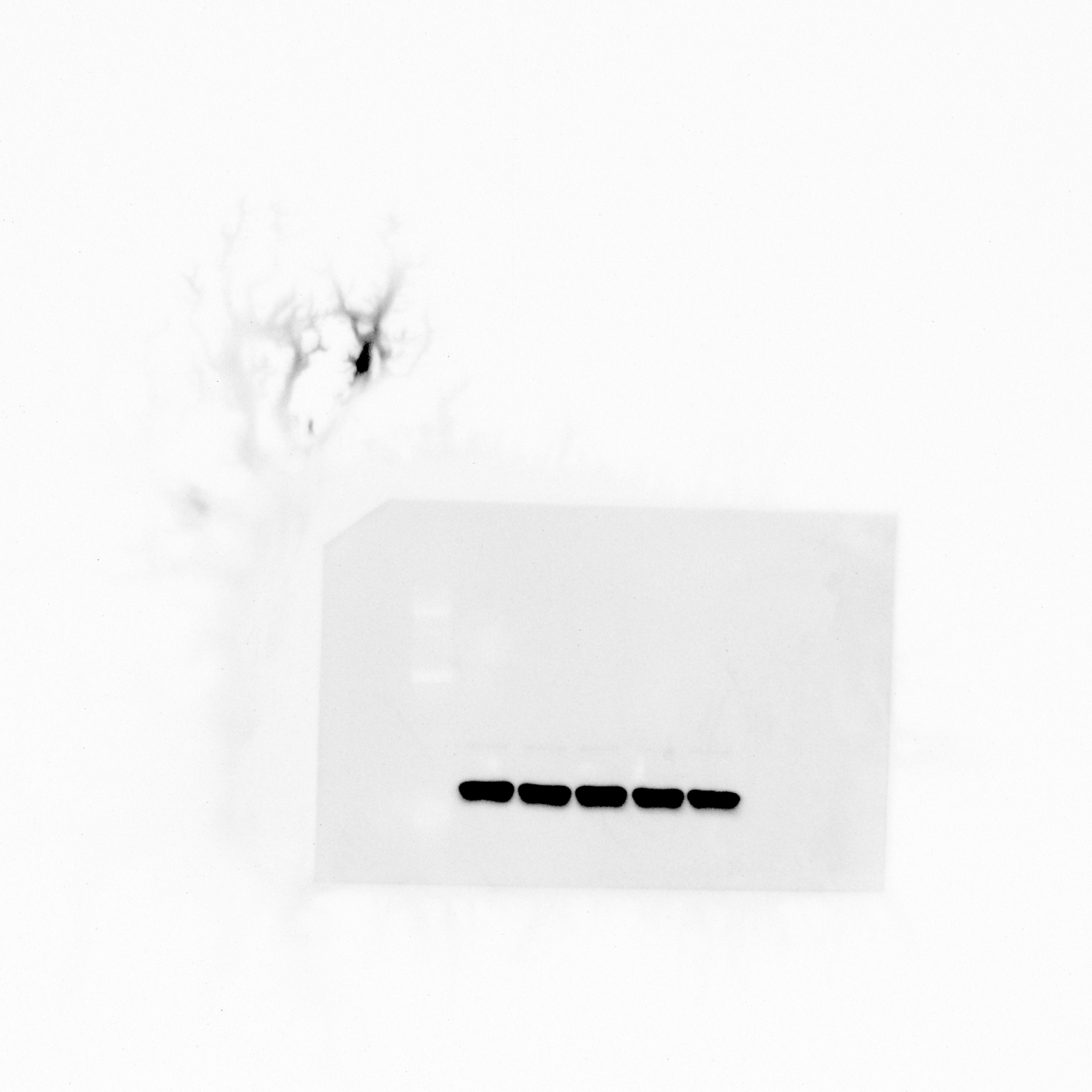


Bax-mice


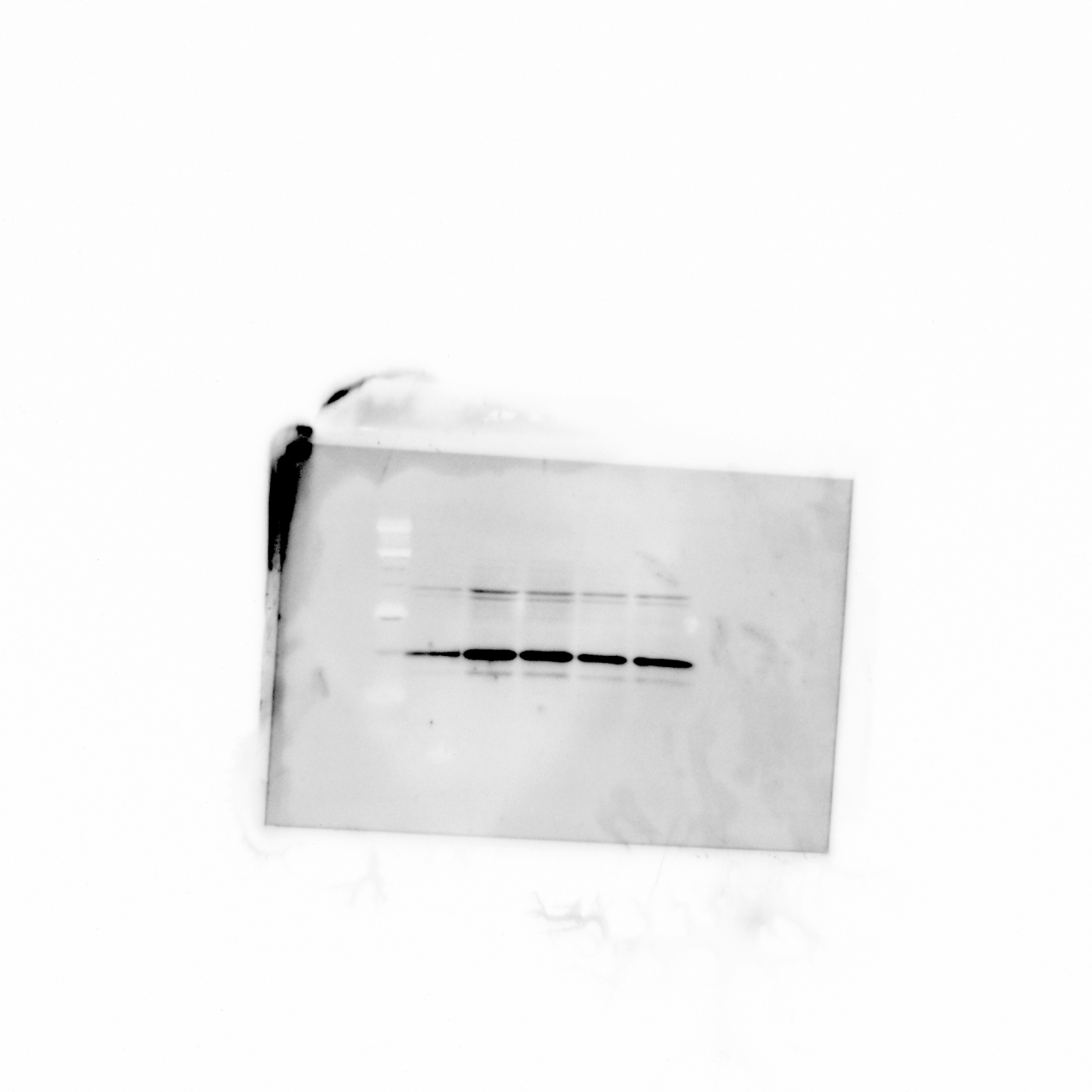


Bcl-2-mice


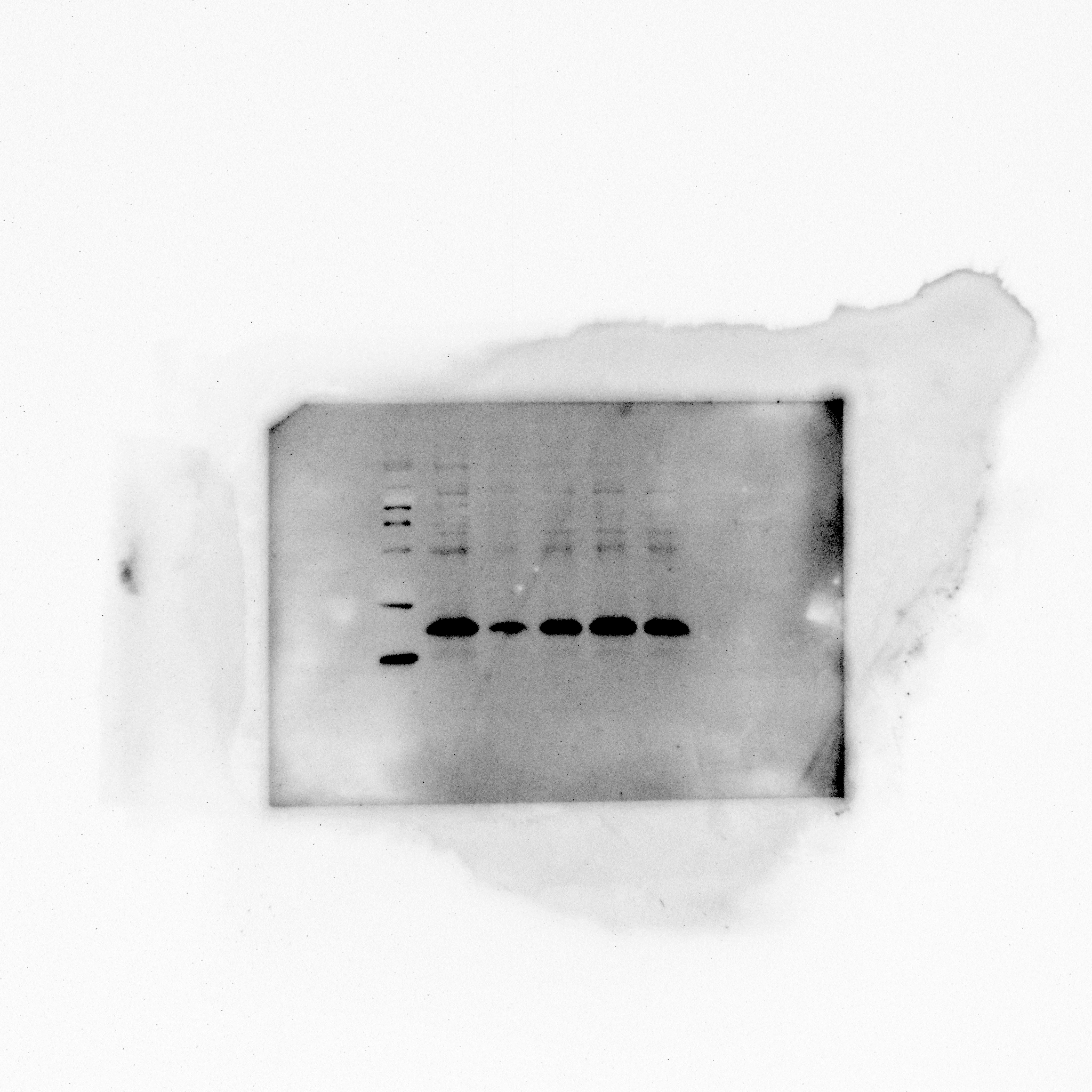


NF-kB-mice


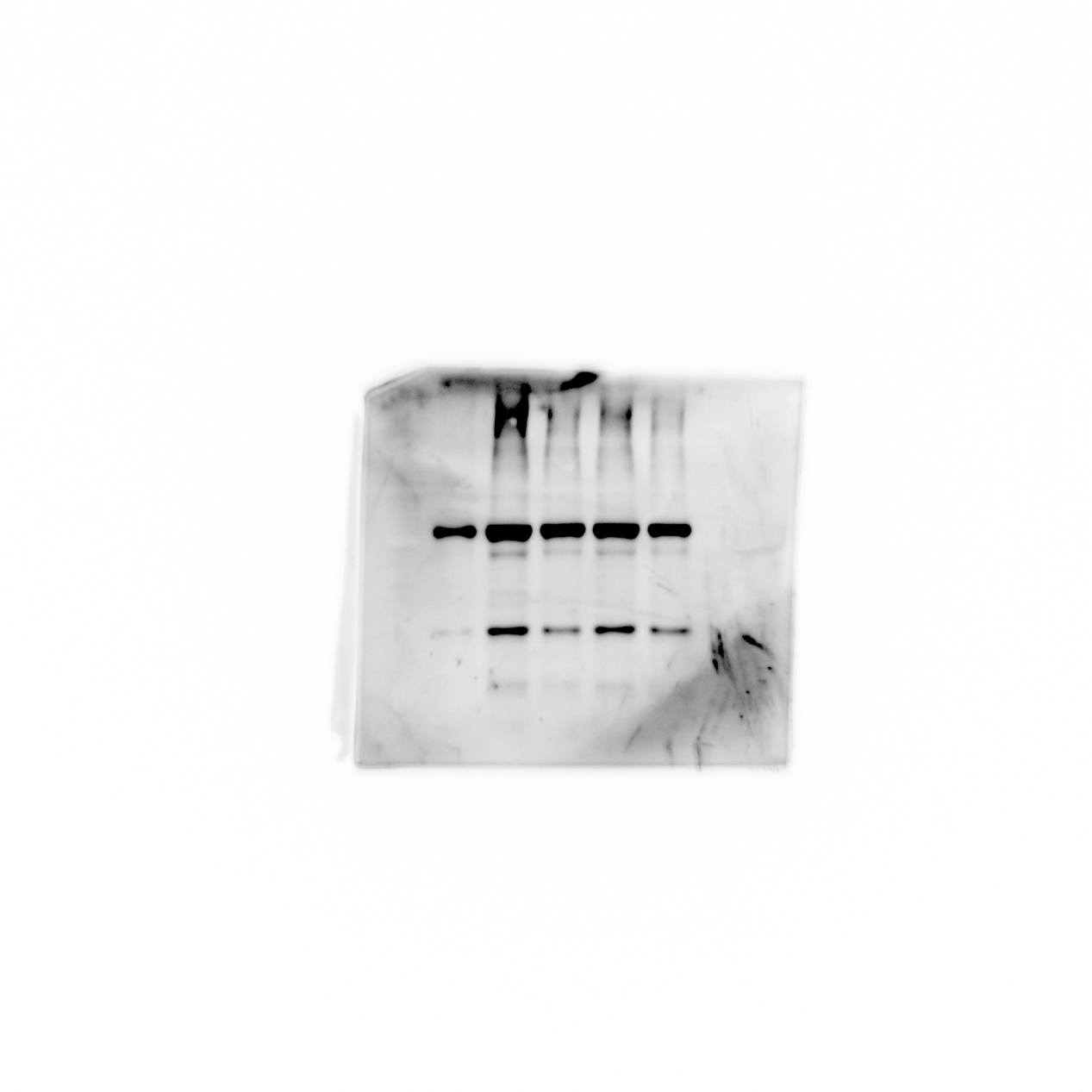


GAPDH-mice


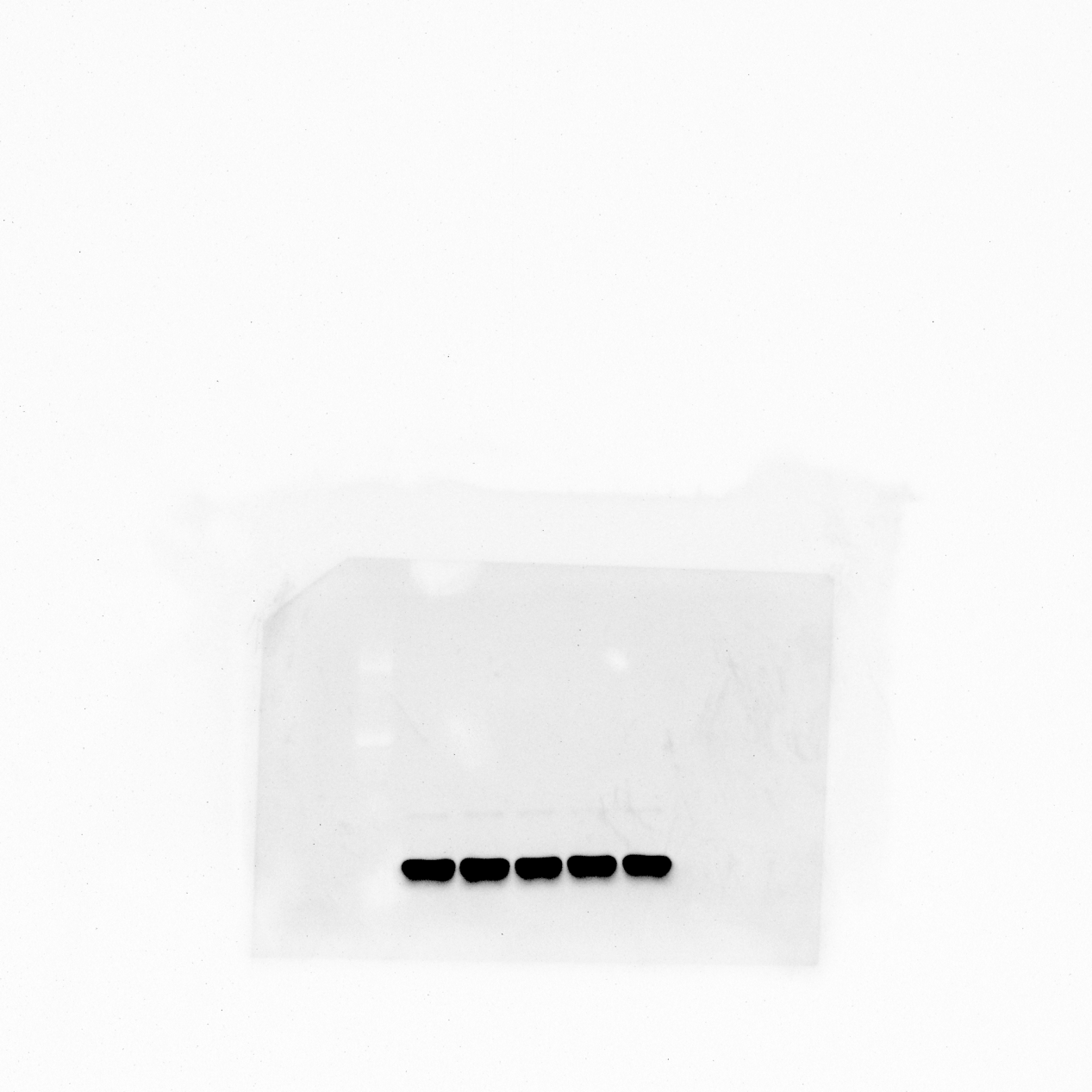


TSE101-2


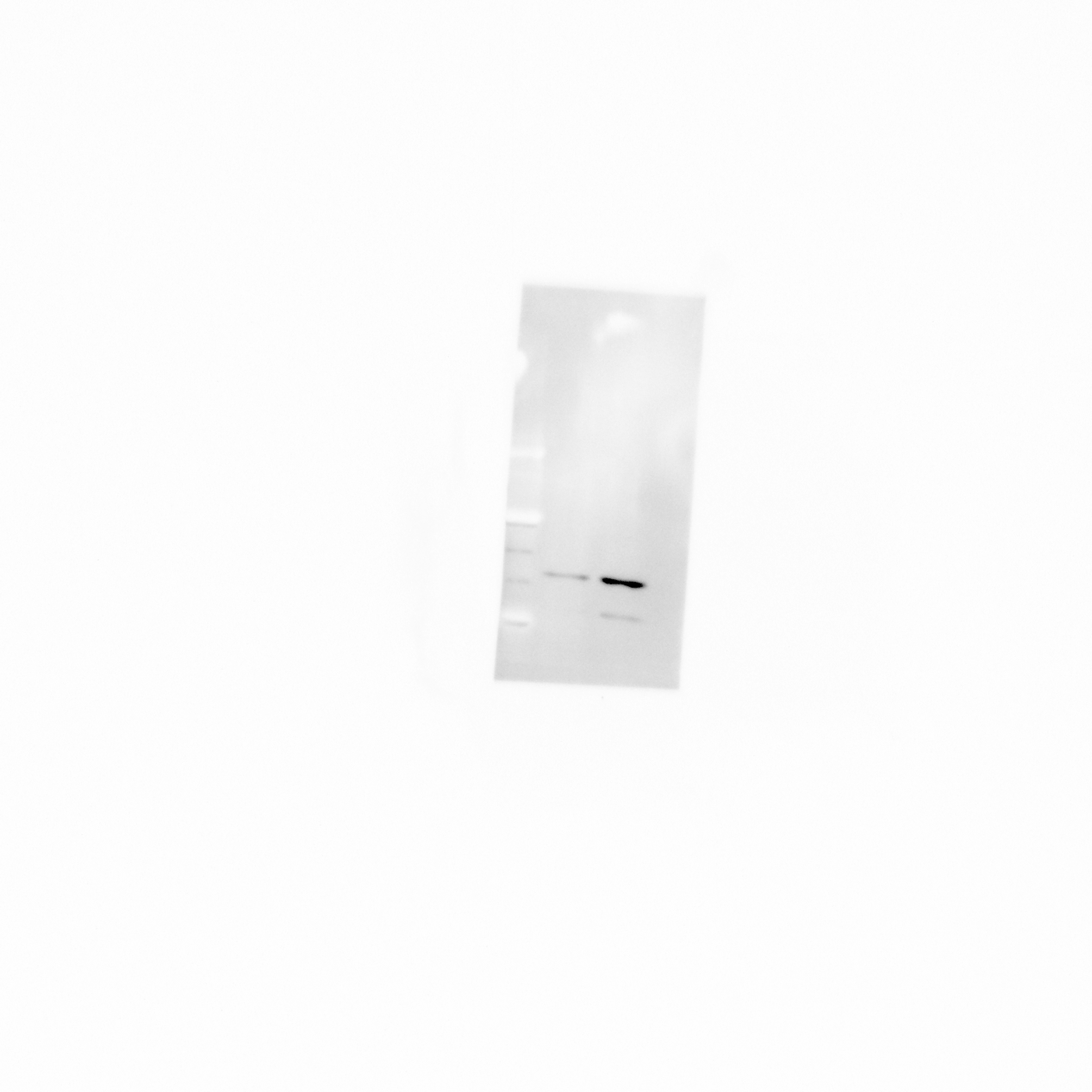


CD63-2


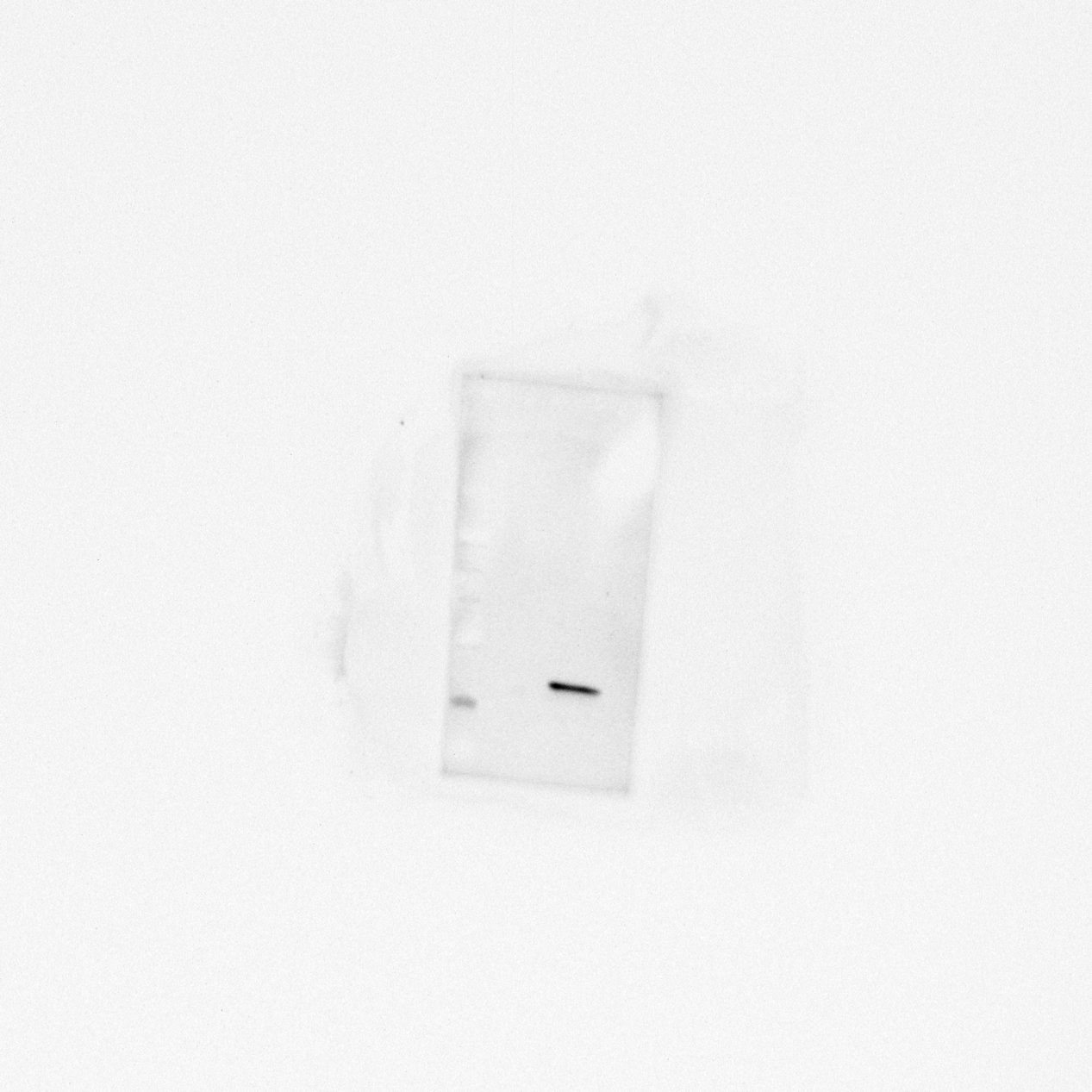


CD81-2


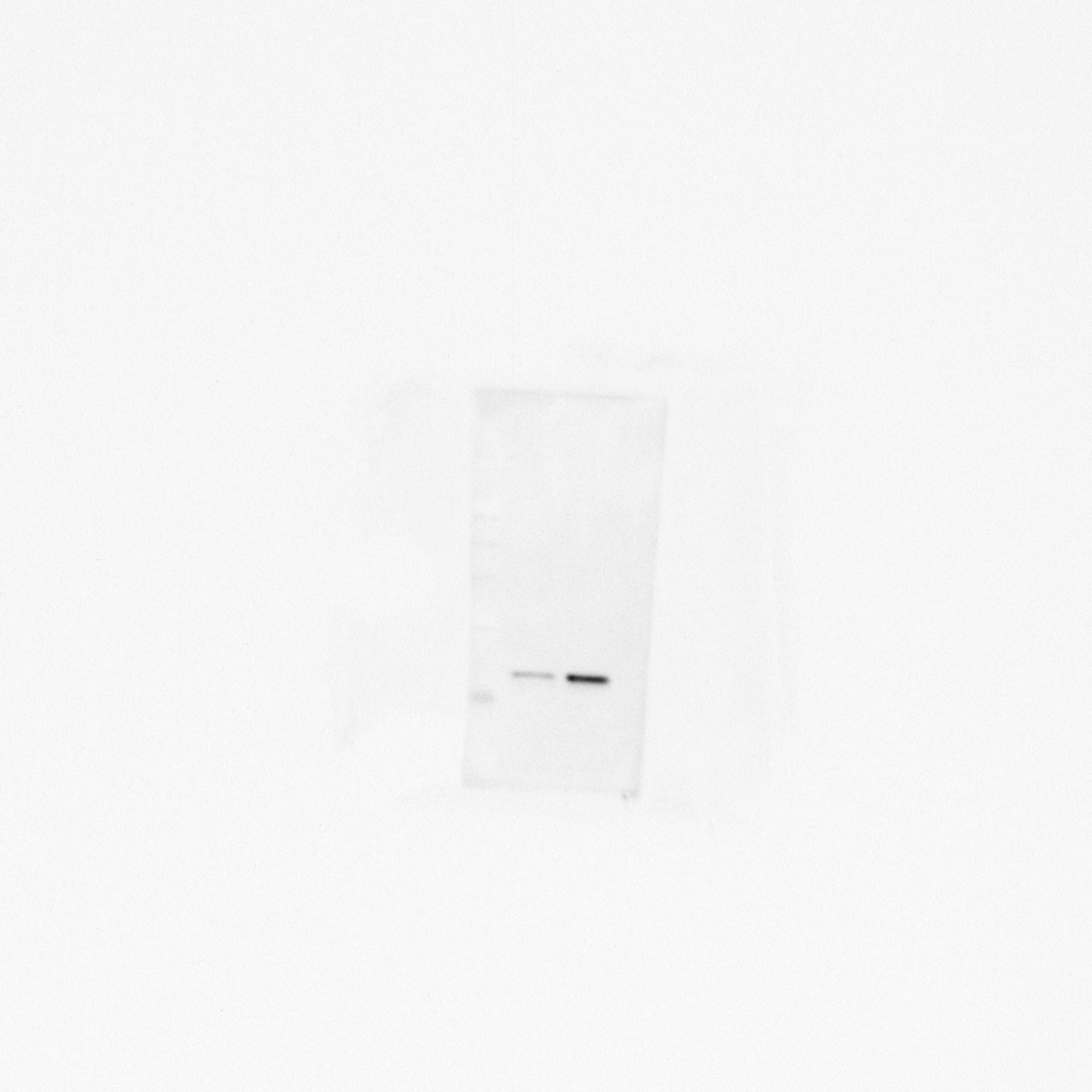

Supplement: Supplementary file 1 — Supplementary Material 1 [file 41598_2026_50402_MOESM1_ESM.docx]
